# Moderate acute alcohol use impairs intentional inhibition rather than stimulus-driven inhibition

**DOI:** 10.1007/s00426-020-01353-w

**Published:** 2020-05-19

**Authors:** Yang Liu, Raoul P. P. P. Grasman, Reinout W. Wiers, K. Richard Ridderinkhof, Wery P. M. van den Wildenberg

**Affiliations:** 1 Department of Psychology, University of Amsterdam, Amsterdam, The Netherlands; 2 Addiction, Development, and Psychopathology (ADAPT) Lab, Department of Psychology, University of Amsterdam, Amsterdam, The Netherlands; 3 Amsterdam Brain and Cognition (ABC), University of Amsterdam, Amsterdam, The Netherlands

## Abstract

Moderate alcohol intake may impair stimulus-driven inhibition of motor actions in go/no-go and stop-signal tasks. Exposure to alcohol-related cues has been found to exacerbate this impairment. By contrast, the effect of alcohol use on intentional inhibition, or the capacity to voluntarily suspend an action, has rarely been investigated. We examined whether and how moderate alcohol intake affects stimulus-driven inhibition (stop-signal task) and intentional inhibition (chasing bottles task), during exposure to alcohol-related stimuli. One hundred and eleven participants were randomly assigned to an alcohol (male: 0.55 g/kg, female: 0.45 g/kg), placebo, or control group. For the stop-signal task, ANOVAs were performed on stop-signal reaction time (SSRT) and go RT with Pharmacological and Expectancy Effects of Alcohol, Stimulus Category (alcohol-related or neutral), and Sex as factors. For the chasing bottles task, multilevel survival analysis was performed to predict whether and when intentional inhibition was initiated, with the same factors. For the stop-signal task, Sex moderated the Pharmacological Effect of Alcohol on SSRT: only for females, alcohol consumption shortened SSRT. In the non-alcohol groups, males had shorter SSRT than females. Concerning intentional inhibition, the alcohol group initiated intentional inhibition less often, especially when stimuli were non-alcohol related. These findings indicate that (1) stimulus-driven inhibition and intentional inhibition reflect different aspects of response inhibition; (2) moderate alcohol intake negatively affects intentional inhibition (but not stimulus-driven inhibition). Speculatively, the observed impairment in intentional inhibition might underlie the lack of control over alcohol drinking behavior after a priming dose. This study highlights the potential role of intentional inhibition in the development of addiction.

**Electronic supplementary material:**

The online version of this article (10.1007/s00426-020-01353-w) contains supplementary material, which is available to authorized users.

## Introduction

Studies on the relation between acute alcohol use and response inhibition have focused exclusively on exogenously driven inhibition. This refers to situations in which the stop process is triggered by an external no-go or stop signal (presented in the context of a go/no-go task or stop-signal task, respectively). Endogenously driven inhibition, on the other hand, has rarely been investigated. Here, inhibition is instigated intentionally, without external stimulus. The current study reports a double-blind, placebo-controlled investigation of the acute effects of alcohol intake on these two qualitatively different types of inhibitory control over behavior, namely stimulus-driven inhibition versus intentional inhibition.

### Acute alcohol use and stimulus-driven inhibition

There is abundant research on the acute effects of alcohol on response inhibition using the go/no-go task (Donders, 1969) and the stop-signal task (Logan, 1994). Compared with the go/no-go task, which primarily measures inhibition errors, the stop-signal task provides an estimate of the latency to stop an initiated action after the presentation of an external stop signal (stop-signal reaction time, SSRT). It was found that moderate to high doses of alcohol (ranging from 0.4 to 0.8 g/kg) lengthened SSRT compared to placebo and control conditions (see Table S1a for an overview of relevant studies), although findings are mixed (e.g., Loeber & Duka, 2009). A rather recent line of research further showed that including alcohol-related stimuli in the task impaired inhibitory control in heavy/dependent alcohol users even when they were sober (for a meta-analysis, see Jones, Duckworth, Kersbergen, Clarke, & Field, 2018). Given these findings, it is likely that inhibitory control deficits caused by acute alcohol use are exacerbated during exposure to alcohol-related stimuli (Field, Kiernan, Eastwood, & Child, 2008). A directly relevant study examined the acute effect of alcohol intake on stopping in male problem drinkers using a lexical stop-signal task (Zack et al., 2011). Male participants classified words and non-words presented on a computer screen with a left- vs. right-hand button press. The results show that SSRT prolonged gradually from the control group to the placebo and alcohol groups (0.7 g/kg), irrespective of word category (alcohol-related vs. neutral words). Additionally, a stress manipulation moderated the effect of word category on stopping. That is, only under stress, alcohol-related words induced longer stopping. Given the male sample, the conclusions could not be generalized to females, who might show different effects than males (Fillmore & Weafer, 2004; Quinn & Fromme, 2016; Weafer & Fillmore, 2012). To examine the generalization of these findings to females, we administered a similar lexical stop-signal task to a larger sample with a similar number of females and males, without the stress manipulation.

### Intentional inhibition

In the go/no-go task and the stop-signal task, external cues trigger the inhibition process. The capacity to decide internally to inhibit an action without any external instruction comprises another important aspect of self-control. Intentional inhibition has been defined as the capacity to voluntarily suspend or inhibit an about-to-be-executed action at the last moment (Filevich, Kühn, & Haggard, 2012). It recruits cortical mechanisms partially distinguishable from those characterizing stimulus-driven inhibition (Kühn, Haggard, & Brass, 2009). The impairment of intentional inhibition occurs in several clinical disorders such as attention deficit hyperactivity disorder (ADHD), addiction, and certain personality disorders (Kühn et al., 2009). With respect to acute alcohol use, a priming dose of alcohol triggers craving and further alcohol-seeking behavior, which likely occurs in a typical drinking occasion (Field, Wiers, Christiansen, Fillmore, & Verster, 2010).

Over the years, there have been several attempts to study intentional inhibition with paradigms such as the marble task (Kühn et al., 2009), and modified go/no-go tasks (Parkinson & Haggard, 2014). These tasks adopted a “free choice” design, where participants could freely decide on which trials to inhibit/go, with an average inhibition rate close to 50%. However, the methodology of these studies is acknowledged to be suboptimal. First, the possibility of pre-decision cannot be ruled out. Hence, the decision to trigger inhibition is not necessarily made on the spot. Second, participants’ choices are somewhat arbitrary. Typically, their decision to inhibit or not does not entail any consequences. To mitigate these limitations, we developed the Chasing Memo task, in which participants can freely decide when to disengage from visuomotor tracking (Liu et al., 2020). Our previous study has indicated that past-year alcohol use did not predict intentional stop-tracking times; comparable behavioral patterns were found after alcohol administration. Three main modifications were made for the present task. First, inspired by the www-model of intentional action (Brass & Haggard, 2008), we designed our task such that it separates the whether and when components of intentional inhibition. According to the www-model, intentional inhibition should include three components of what, when and whether. These components are partially independent at the cognitive as well as the neural implementation level (Zapparoli, Seghezzi, & Paulesu, 2017; Zapparoli et al., 2018). We, therefore, distinguished ‘whether to inhibit’ from ‘when to inhibit’ to examine potential differential effects of alcohol on these distinct components. Second, beverage bottles were embedded to increase ecological validity. In this way, the task was renamed as the chasing bottles task. Third, the decision of whether to continue or to stop tracking yielded different rewards. To continue tracking led to immediate reward (cf. the instant pleasure from drinking), whereas to stop tracking was associated with higher future reward. The idea of delayed gratification (i.e., resist the temptation of an immediate reward in preference of a delayed reward, Mischel, Shoda, & Rodriguez, 1989) was used here.

### The present study

The current experiment conforms largely to previous experimental designs. As shown in Table S1a, studies of acute alcohol use and response inhibition usually included an alcohol group and a placebo group. This allows examining the pharmacological effect of alcohol as both groups expected alcohol delivery. To also explore the expectancy effect of alcohol on inhibitory control, we added a control group. A fully balanced placebo design was not adopted, because the anti-placebo group (i.e., drink alcohol but expect non-alcoholic beverage) is difficult to realize (Martin & Sayette, 1993).

The primary goal of the present study was to examine the effect of a moderate dose of alcohol on both stimulus-driven and intentional inhibition, measured respectively by a lexical stop-signal task and the chasing bottles task. The two computer tasks included a condition with alcohol-related stimuli to examine the effect of appetitive cues and their interaction with alcohol intoxication on inhibition performance. Equal numbers of males and females were recruited for a moderation effect test. In addition, questionnaires to assess substance use, response to alcohol, impulsivity, and reward sensitivity were administered for evaluating potential between-group differences and/or their associations with task performance. We hypothesized that: (1) alcohol intake impairs both stimulus-driven and intentional inhibition; (2) these alcohol-related effects are stronger in a context with alcohol-related stimuli compared to non-alcohol related stimuli; (3) sex moderates the relationship between the group and task performance, with males being more influenced by alcohol; (4) laboratory measures of response inhibition (i.e., stop-signal task performance, chasing bottles task performance) should be weakly associated with self-reported impulsivity.^1^

## Methods

### Participants

A total of 111 participants were recruited, mostly university students. Inclusion criteria were: (1) aged between 18 and 25, (2) weight between 50 and 100 kg, (3) not alcohol-naïve and no alcohol dependency, identified by an AUDIT (Alcohol Use Disorder Identification Test, Saunders, Aasland, Babor, de la Fuente, & Grant, 1993) score between 5 and 16, (4) daily cigarettes < 4, (5) fluent in Dutch, (6) not on any medication, (7) normal or corrected to normal eyesight, (8) no diagnosis of neurological problems including epilepsy, head trauma, (9) Beck Depression Inventory for Primary Care score < 5 (Beck, Guth, Steer, & Ball, 1997). Five participants were excluded: three participants in the placebo group who did not believe that they drank alcohol, one participant with a baseline Breath Alcohol Concentration (BrAC) level above zero, and one participant tested positive for Tetrahydrocannabinol (i.e., the principal psychoactive constituent of cannabis). The remaining 106 participants were matched on demographics, gender ratios, alcohol and other substances’ use (Table 1).

**Table 1:** Group comparison: demographics and substance use (*N* = 106)

Variables	Alcohol (*n* = 33)	Placebo (*n* = 36)	Control (*n* = 37)
	*M* (SD)	*M* (SD)	*M* (SD)
Age	21.12 (1.92)	21.06 (2.03)	21.17 (1.91)
Sex (M/F)	17/16	18/18	18/19
AUDIT	8.91 (2.74)	9.14 (3.00)	9.30 (3.14)
Alcohol use last month			
Drinking days (weekdays)	4.73 (3.29)	4.88 (3.82)	4.80 (2.79)
Drinking days (weekend)	4.00 (2.19)	4.11 (2.50)	4.14 (2.29)
Drinks per occasion (weekdays)	3.79 (2.52)	3.42 (2.42)	3.84 (2.65)
Drinks per occasion (weekend)	5.20 (2.25)	4.40 (1.81)	4.86 (2.88)
Smoker/non-smokers	5/28	6/30	6/31
Daily cigarette	2.2 (0.84)	2.0 (0.89)	2.0 (0.83)
Other substances (times last month)			
Marijuana	1.36 (2.26)	0.43 (1.42)	1.49 (3.70)
Cocaine	0.15 (0.87)	0	0
Ecstasy	0.30 (1.21)	0.14 (0.85)	0
Club drugs	0.15 (0.87)	0.29 (1.18)	0.14 (0.82)

Substances use was measured by the Core Alcohol and Drug Survey (CORE, see Supplementary Materials). Except for the four substances listed here, the usage of other substances was very rarely reported

### Modified stop-signal task

A lexical stop-signal task based on the study by Zack et al. (2011) was used to test stimulus-driven inhibition. Participants were instructed to press corresponding buttons (‘z’ vs. ‘/’) to distinguish between actual words and non-words that were presented on a computer screen. A change in font color from grey to red indicated the external stop signal, which occurred on 25% of the trials. The actual words were selected by a separate group of participants, with length and frequency matched to the alcohol-related words. Forty alcohol-related words (in Dutch), 40 neutral words, and 80 non-words were selected and used in the testing stage (Table S2). Each trial started with a fixation screen for 500 ms, followed by a letter string that disappeared upon response or until 1500 ms had elapsed. Then a jittered inter-trial interval (ITI) followed, ranging from 1250 to 1750 ms in steps of 50 ms. The initial stop-signal delay (SSD) was 200 ms, and increased/decreased by 50 ms, respectively, after a successful/failed inhibition. This dynamic tracking procedure was adopted across blocks and for each word category separately. The testing stage consisted of a practice block and five equivalent experimental blocks. Each block consisted of 160 trials with all the selected stimuli in a random sequence without repetition. To practice the lexical decision task, a familiarization block (96 trails) with different stimuli was administered before drinking. The stop-signal task took about 40 min to complete. SSRT, go RT (mean reaction time of correct go trials), stop rate and SSD were recorded. SSRT was calculated by subtracting mean SSD from the *n*th go RT (i.e., the rank-ordered go RT that corresponds to the percentage of failed inhibition, see integration method: Logan & Cowan, 1984). Longer SSRT indicates prolonged inhibition latency.

### Chasing bottles task

**Fig. 1:**
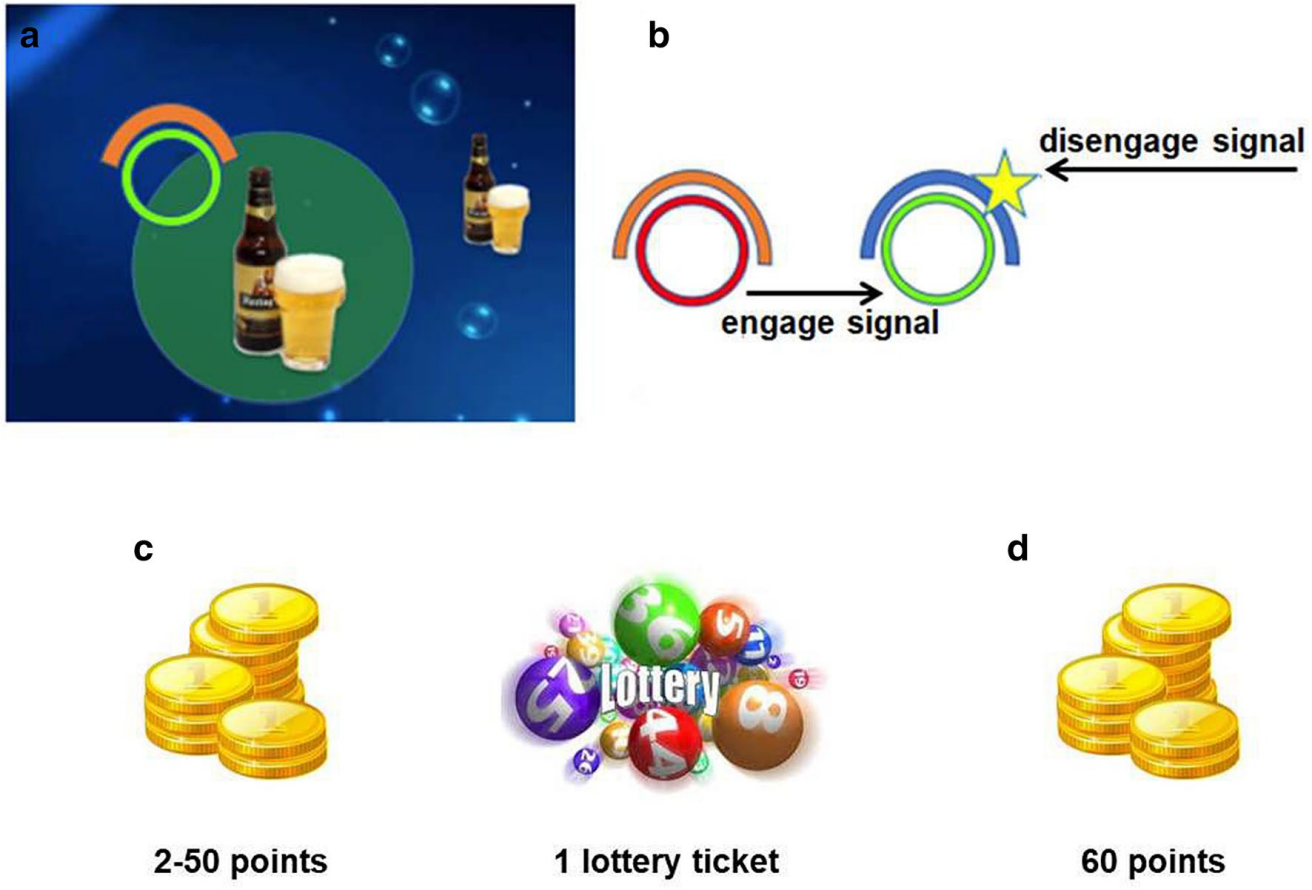
**a** The screen background and layout of the Chasing Bottle task. Participants move the mouse and keep the cursor within the green zone in order to track the floating bottle. **b** The engage and disengage signal of the task. When the red circle turned to green, participants should start tracking. The appearance of the star signaled the beginning of a 20-s time window, within which participants can stop tracking if they felt the urge to do so. Otherwise, they can continue tracking until 20 s has elapsed and the trial reached its end automatically. **c** If the participant stopped tracking within the 20-s window, the feedback for that trial included a lottery ticket (related to the possibility of winning a voucher in the future) and a random number of points (between 2 and 50, related to the extra payment). **d** If the participant did not stop tracking within the 20-s window, they always got 60 points

In this intentional inhibition task, a bottle moved (‘floated’) at 9.5 cm/s against the background of the bottom of an ocean, changing directions at random angles between 0° and 115° and at intervals between 556 and 1250 ms. The participants’ main task was to track the bottle by moving the computer mouse and to keep a yellow dot within a green zone of 2 cm radius (Fig. 1a, b). A smaller bottle preceded the target bottle to indicate its course and to facilitate the ease and accuracy of tracking. A circle at the top left corner of the green zone served as the go signal (from red to green). After uninterrupted successful tracking for 2 s, a yellow star was displayed, which signaled the onset of a 20 s window. During this period, participants can stop tracking if they felt the urge to do so, or continue tracking to the end of a trial. Two counters presented reward feedback. For a stopped trial, participants earned points and a lottery ticket (Fig. 1c). The number of points was a random number between 2 and 50. Stopping too early or too late was associated with only 2 points and was discouraged. For a non-stopped trial, participants always earned 60 points (Fig. 1d). The points accumulated were converted into payment with the ratio of 1500:1 on the spot. The accumulated lottery tickets were associated with the chance of winning a €10 voucher upon project completion. Participants were instructed and trained to follow their urge to stop rather than preplan or use external cues (e.g., the spatial position of the bottle). They were also instructed that some variability of tracking latency and decision to disengage/engage was monetary beneficial. A plastic bottle without brand was used in the familiarization stage. Two categories of bottles (alcoholic vs. non-alcoholic beverages, see Fig. S1) were used in the testing stage with valence, arousal, and urge values matched (Pronk, van Deursen, Beraha, Larsen, & Wiers, 2015). The testing stage included 6 blocks, with 10 trials each. There was no repetition of bottles within one block. And stimuli were presented in a randomized order. At the end of this task, bottles were evaluated in terms of valence, arousal, and dominance with a 9-Likert scale. This task took about 35 min to finish.

### Alcohol administration

Blood alcohol concentrations (BAC) of males and females were matched by administering 0.55 g/kg and 0.45 g/kg of alcohol, respectively. The volume of vodka (40% alcohol by volume) was calculated through the following formula (Korucuoglu, Gladwin, & Wiers, 2015).

$${\text{Males}}:{\text{volume}} = {\text{weight}} \times 0.55\frac{{\text{g}}}{{{\text{kg}}}} \div 0.789 \frac{{\text{g}}}{{{\text{ml}}}} \div 40\%$$

$${\text{Females}}:{\text{volume}} = {\text{weight}} \times 0.45\frac{{\text{g}}}{{{\text{kg}}}} \div 0.789 \frac{{\text{g}}}{{{\text{ml}}}} \div 40\% ,$$

where 0.789 g/ml represents the density of ethyl alcohol.

A maximum of 4 standard drinks (one standard drink contains 10 g of pure alcohol in The Netherlands) was administered to females and 5 for males, which was associated with the maximized weight. Alcoholic drinks were prepared with one portion of vodka and three portions of orange juice and divided into three drinks. The placebo drink was prepared with tonic water instead of vodka. To make it smell and taste like alcohol, drinks for the alcohol and placebo groups were prepared with a slice of lemon soaked in vodka, vodka sprayed on the rim of the glass and three drops of Tabasco sauce (McIlhenny Co., USA) on the top (Korucuoglu, Gladwin, & Wiers, 2016). The control group drank tap water.

### Procedure

Participants were instructed to refrain from alcohol intake (24 h), other substances (1 week), smoking (4 h), caffeine-containing drinks (4 h) and big meals (4 h) prior to the experiment. The tests took place between noon and 7 p.m.

**Fig. 2:**
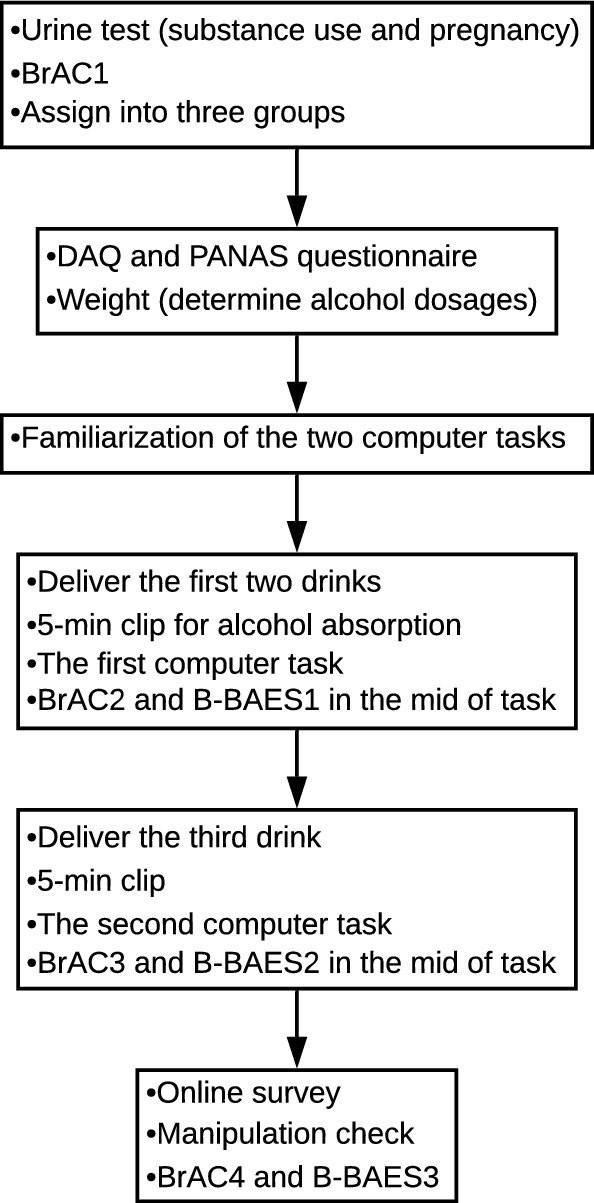
The procedure of the experiment. *BrAC* Breath Alcohol Concentration, *DAQ* Desire for Alcohol Questionnaire, *PANAS* Positive and Negative Affect Scale, *B-BAES* Brief Biphasic Alcohol Effects Scale, the online survey included SRE (Self-Rating of the Effects of Alcohol), RAPI (Rutgers Alcohol Problem Index), DII-short (Dickman’s impulsivity inventory short-version), SPSRQ (Sensitivity to punishment and sensitivity to reward questionnaire) and frequency of alcohol and binge drinking

As summarized in Fig. 2, the experiment consists of six mini parts. First, upon arrival in the lab, a urine test and a baseline BrAC (by Alcoscan ALC-1) were performed to exclude past week substance use, pregnancy (females) and alcohol use. Participants were then randomly assigned to one of three groups (i.e., alcohol, placebo or control). Second, participant weight was measured to determine alcohol dosage to be administered. The Desire for Alcohol Questionnaire (Love, James, & Willner, 1998) and the Positive and Negative Affect Scale (Watson, Clark, & Tellegen, 1988) were tested prior to alcohol administration as craving for alcohol and mood are likely to be influenced by intoxication (detailed information about all questionnaires is available in Supplementary Online Materials S1). Third, participants were familiarized with the computer tasks by explanation and practice with different task stimuli. Fourth, the first two drinks were served continuously. Each had 3 min to finish and 2 min for mouth-wash. Next, a 5-min short video clip was played before performing the first computer task to allow alcohol absorption (Korucuoglu, Gladwin & Wiers, 2015). Fifth, the third top-up drink was delivered, followed by another short clip and the second computer task. The order of the two computer tasks was counterbalanced across participants. Sixth, an online survey was administered, including the Self-Rating of the Effects of Alcohol (Schuckit, Smith, & Tipp, 1997), the Rutgers Alcohol Problem Index (White & Labouvie, 1989), the Dickman’s Impulsivity Inventory (Dickman, 1990), the Sensitivity to punishment and sensitivity to reward questionnaire (SPSRQ, Torrubia, Ávila, Moltó, & Caseras, 2001), and the manipulation check question “How much alcohol do you think you have had?”. BrAC and the Brief Biphasic Alcohol Effects Scale (B-BAES, Rueger, McNamara, & King, 2009) were measured sequentially three times across the session (i.e., mid of the two computer tasks and at the end of the experiment). The procedure for the control group was almost the same, except that BrAC was only tested at baseline, B-BAES and the manipulation check question were omitted. The whole experiment took about 2 h and 15 min to complete.

Participants received 20 euro or 2 course-credits, and up to €2.50 extra payment based on their performance in the chasing bottles task. The study was approved by the local ethics committee and complied with the 1989 Helsinki Declaration.

### Statistical analysis

#### Questionnaires

BrAC was analyzed within the alcohol group, with a paired *t* test to compare values during different tasks and a repeated-measures ANOVA to assess the interaction between Sex and Time Points (BrAC_2_ and BrAC_3_).^2^ Repeated-measures ANOVAs were performed for the Brief Biphasic Alcohol Effects stimulant and sedative subscales, with Group (alcohol/placebo) and Time Points as factors. Other questionnaires (e.g., alcohol sensitivity, impulsivity, reward sensitivity) were compared between groups by one-way ANOVAs (see analyses and results in Supplementary Online Materials **S1**).

#### Stop-signal task

SSRT, go RT, and stop rate were analyzed with 2 Word Category (alcohol- vs. non-alcohol words) × 2 Sex × 2 Consume Alcohol (yes/no) × 2 Expectancy of Alcohol (yes/no)^3^ repeated-measures ANOVAs. In line with Zack et al. (2011), the performance on the non-words was analyzed with similar ANVOAs without the factor of Word Category. In a secondary analysis, the correlation coefficients between SSRT and self-reported impulsivity were calculated by averaging Fisher's *Z* of correlation coefficient of each group and transforming back (Silver & Dunlap, 1987).

#### Chasing bottles task

A mixed-effect survival analysis was performed on this time-to-event data (Ferreira & Patino, 2016; Sloan et al., 2019) with participant as random factor in R 3.4.4 (coxme package: Therneau & Lumley, 2015). Here the event is disengagement from tracking, which will be censored if the participant did not stop tracking within the 20-s time window. The model included the main effect of bottle category, consume alcohol, their interactions, the stratification variable expectancy of alcohol and sex,^4^ and a random subject (‘frailty’) term to account for inter-individual differences. In a secondary analysis, SSRT, self-reported impulsivity, and reward sensitivity were used to predict stopping probability, one at a time. Total reward (i.e., the number of points and lotteries) from this task was not considered as a main outcome for analysis as it was aimed to balance the ratio of inhibited and non-inhibited trials, and meanwhile simulate the reason-responsive nature of intentional inhibition (Haggard, 2018). Furthermore, the employment of volition rather than strategies in maximizing reward was emphasized to the participants when performing this task, which renders the amount of reward less informative in explaining intentional inhibition behavior.

## Results

### BrAC

BrACs were comparable across the two tasks [stop-signal task: *M* = 0.39 ‰, SD = 0.10 ‰, chasing bottles task: *M* = 0.39 ‰, SD = 0.11 ‰, *t*(32) = 0.06, *p* = 0.96]. The interaction between sex and time points revealed a main effect of time points [*F* (1, 31) = 77.08, *p* < 0.01]. BrAC_2_ was lower than BrAC_3_ (*M* = 0.34 ‰, SD = 0.09 ‰ vs. *M* = 0.44 ‰, SD = 0.09 ‰, Fig. S3). This was controlled by adding task sequence as a covariate in the main analyses of both tasks. The main effect of sex [*F* (1, 31) = 0.31, *p* = 0.58] and its interaction with time points were not significant [*F* (1, 31) = 1.40, *p* = 0.25].

### Manipulation check

There are two measures related to the manipulation check, namely perceived alcohol consumed and the Brief Biphasic Alcohol Effects Scale. First, participants in the alcohol group thought they drank more alcohol (*M* = 3.88, SD = 1.55, range 1–10) than those in the placebo group [*M* = 2.04, SD = 1.50, range = 0–5, *t*(71) = 5.20, *p* < 0.01], which was expected (Testa et al., 2006). Importantly, perceived alcohol contents in both groups were significantly above 0 (*p*s < 0.01), validating the placebo manipulation. Second, the stimulant subscale of the Brief Biphasic Alcohol Effects Scale revealed a main effect of time point [*F* (2, 142) = 20.16, *p* < 0.01, *η*^2^ = 0.22, Fig. S4a]. It declined on average 0.80 from the mid of the first task to the mid of the second task (*p* < 0.01), followed by an averaged decline of 0.10 at the end of the experiment (*p* = 0.73), indicating subjects felt less stimulated as the session proceeded. The main effect of group (alcohol vs. placebo) was not significant [*F* (1, 71) = 2.96, *p* = 0.09], nor was its interaction with time point [*F* (2, 142) = 1.10, *p* = 0.35]. The sedative subscale also revealed a main effect of time points [*F* (2, 142) = 3.95, *p* = 0.02, *η*^2^ = 0.05, Fig. S4b). It increased by an average of 0.06 from the mid of the first task to the mid of the second task (*p* = 0.70), followed by a reduction of 0.43 at the end of the experiment (*p* < 0.01). The main effect of Group was also significant [alcohol: 5.17 (1.86), placebo: 4.20 (1.92), *F* (1, 71) = 5.87, *p* = 0.02, *η*^2^ = 0.08], indicating subjects felt more sedated after alcohol than after placebo. The interaction between time point and group was non-significant [*F* (2, 142) = 0.02, *p* = 0.98].

### Stop-signal task

**Fig. 3:**
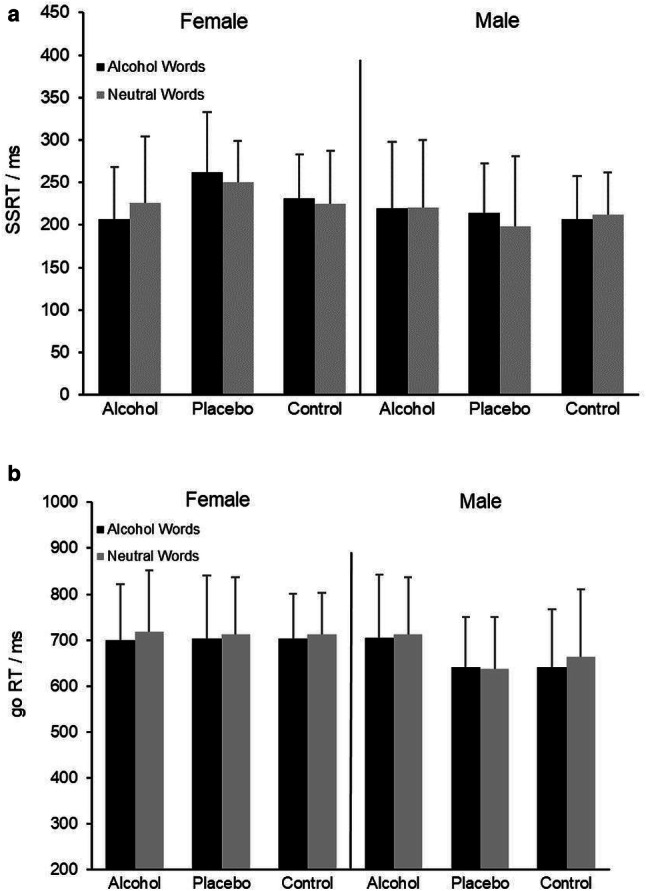
**a**, **b** Mean stop-signal reaction time (SSRT) and go RT to alcohol-related words and neutral words per group, separately for males and females

The repeated-measures ANOVA of SSRT revealed a significant interaction between Consume Alcohol and sex [*F* (1, 99) = 5.02, *p* = 0.03, *η*^2^ = 0.05, see Fig. 3a]. Post hoc analyses were performed by splitting the data set on these two factors. These analyses revealed that females who drank alcohol had significantly shorter SSRT than females who did not drink alcohol [216 ms (70) vs. 242 ms (60), *F* (1, 49) = 5.67, *p* = 0.02, *η*^2^ = 0.10]. No significant difference was found amongst males [220 ms (78) vs. 208 ms (61), *F* (1, 49) = 0.52, *p* = 0.47]. For those who did not have alcohol (i.e., placebo and control), males had shorter SSRT than females [208 ms (61) vs. 242 ms (60), *F* (1, 68) = 9.76, *p* =  < 0.01, *η*^2^ = 0.12]. Such a gender difference was absent in the alcohol group [male: 216 ms (70) vs. female: 220 ms (78), *F* (1, 30) = 0.06, *p* = 0.81]. No main or interaction effects were significant regarding go RT (Fig. 3b) and stop rates. Test statistics are reported in Table S3a–S3c. As to the non-words, no main effect of consume alcohol, expectancy of alcohol, sex, task sequence, nor their interactions were significant concerning SSRT, go RT, and stop rates (see Table S4a–S4c for test statistics).

### Chasing bottles task

**Fig. 4:**
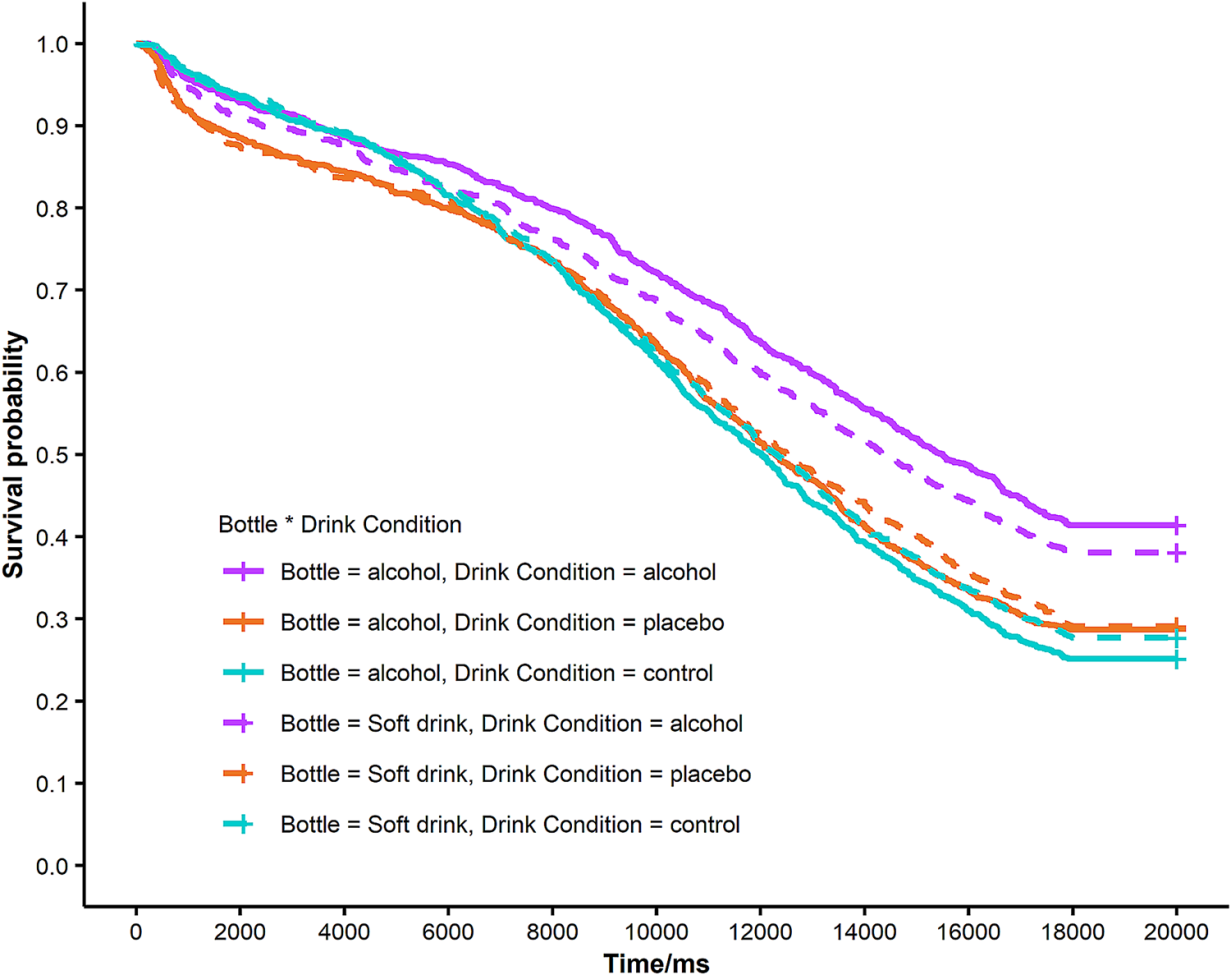
Survival curves for each drink condition per bottle category. People who drank alcohol generally stopped less frequently compared with those in the placebo group and the control group. The placebo and the control group showed similar stopping probability as the survival lines almost overlap, especially from 8 s on. For the alcohol group, people were less willing to stop tracking soft drink bottles than alcohol bottles

Five additional participants were excluded from the analysis as they inadvertently received different instructions (i.e., the ratio of immediate and delayed reward differed from the remaining participants). The main effect of Consume Alcohol was significant, with alcohol consumption decreasing the disengagement rate by approximately 32% over the course of a trial [likelihood ratio test: *χ*^2^ (1) = 1343.55, *p* < 0.01, hazard ratio (HR) 0.68, 95% CI (0.61, 0.76)].^5^ The effect of task sequence [*χ*^2^ (1) = − 1275.74, *p* = 1.00, HR 0.91, 95% CI (0.85, 0.96)] and bottle category [*χ*^2^ (1) = 0.01, *p* = 0.94, HR 0.96, 95% CI (0.89, 1.03)] was not significant. The interaction between consume alcohol and bottle category was also significant [*χ*^2^ (1) = 4.49, *p* = 0.03, HR 1.16, 95% CI (1.01, 1.33), Fig. 4]. Post hoc analysis by splitting the data set revealed that (1) participants in the alcohol group stopped more often when tracking alcohol-related bottles compared to soft drink bottles [HR 1.14, 95% CI (1.01, 1.28), *p* = 0.03]; (2) the effect of bottle category on stopping probability was not significant for those who did not drink alcohol [HR 0.96, 95% CI (0.89, 1.03), *p* = 0.22]; (3) compared with those who did not drink alcohol, participants in the alcohol group had lower stopping probability when tracking soft drink bottles [HR 0.60, 95% CI (0.40, 0.90), *p* = 0.01], and (4) the group difference was not significant when tracking alcohol-related bottles [HR 0.72, 95% CI (0.47, 1.08), *p* = 0.12]. The effect of expectancy effect of alcohol on stopping probability is visualized in Fig. S2a. There was no significant difference on stop rate between those that expected and those that did not expect alcohol [for the first 6 s: HR 1.26, 95% CI (0.92, 1.72), *p* = 0.16, for the remaining 14 s: HR 0.69, 95% CI (0.42, 1.14), *p* = 0.15].

### Correlation analyses

SSRTs associated with the conditions that employed alcohol-related words and neutral words were both negatively correlated with functional impulsivity (*r* = − 0.26, *p* < 0.01; *r* = − 0.24, *p* = 0.02, respectively), but unrelated with stopping probability in the chasing bottles task (see statistics in Supplementary Online Materials S2). This indicated that a lower functional impulsivity score was associated with prolonged stimulus-driven stopping latency. Neither impulsivity nor reward sensitivity was a significant predictor of stopping probability in the chasing bottles task.

## Discussion

The current study explored the effect of a moderate dose of alcohol on stimulus-driven inhibition as well as intentional inhibition. For stimulus-driven inhibition, alcohol consumption shortened SSRT in females; and for those who did not drink, males had shorter SSRT than females. Regarding intentional inhibition, participants who drank alcohol were less likely to disengage from tracking compared with participants in the placebo and control conditions, especially when viewing soft-drink bottles. In addition, the expectancy effect of alcohol showed a time-dependent pattern on intentional disengagement rate, namely a decreasing stopping probability after an initial increase. What is more, SSRT was negatively associated with functional impulsivity, but unrelated with stopping probability in the chasing bottles task.

### Acute alcohol use and stimulus-driven inhibition

Opposite to Zack et al. (2011), who reported alcohol consumption prolonged SSRT in a group of male problem drinkers, we failed to find such a main effect in a sample with males and a similar number of females without a problematic drinking history. Specific factors of the present study might explain this difference. To clarify those potential factors, we compiled a list of existing studies where the effect of acute alcohol use on the stop-signal task performance was tested (see Table S1a) and followed up with some preliminary analyses (see results in Table S1b). We compared studies that did find the impairing effect of alcohol on the stop-signal task performance with studies that did not in terms of sample characteristics, task parameters, the dosage of alcohol administered, and the study design. Regarding sample characteristics, gender ratio and typical alcohol use are possible moderators. It is suggested that males are more vulnerable to the acute effect of alcohol than females (Fillmore & Weafer, 2004), and heavy drinkers are hypersensitive to the short-term effect of alcohol compared to light drinkers (Field et al., 2010). However, both assumptions have very limited empirical and theoretical support. As to task parameters, the most relevant one is the modality of the stop signal. It was stated that studies using auditory stop signals report statistically significant differences in SSRT compared to studies using visual stop signals (Guillot, Fanning, Bullock, McCloskey, & Berman, 2010). The underlying reasons remain unclear, except that auditory stop tones are perceived as more intense than visual stop cues (van der Schoot, Licht, Horsley, & Sergeant, 2005). Regarding the dosage of alcohol administered, in principle, a high dose of alcohol was more likely to cause impaired inhibition than a smaller amount (0.8 g/kg vs. 0.4 g/kg, Caswell, Morgan, & Duka, 2013). However, exceptions exist such that even a high dose failed to impair response inhibition (BAC: 0.10%: Guillot et al., 2010; 0.8 g/kg: Dougherty, Marsh-Richard, Hatzis, Nouvion, & Mathias, 2008), and a low dose was sufficient to cause stopping impairment (0.4 g/kg, de Wit, Crean, & Richards, 2000; Nikolaou, Critchley, & Duka, 2013; Reynolds, Richards, & de Wit, 2006). The study design mainly refers to whether alcohol and placebo manipulation is a between-subject or within-subject factor and whether there is a baseline/pre-drink measure. These design options are relevant as individuals differ in their response to alcohol and there is a day-to-day variance of inhibition performance (Campbell, Chambers, Allen, Hedge, & Sumner, 2017). The fact that the three groups were matched in terms of demographics, typical alcohol use, and especially sensitivity to alcohol, made these concerns less vital in our study. Overall, Table S1b revealed that none of these potential factors had a significant effect on research findings (i.e., positive/negative). Therefore, the absence of an alcohol effect on SSRT amongst the population that usually does not drink too much might not readily be attributed to participants’ low typical alcohol use, the use of visual rather than auditory stop signals, the low amount of alcohol administered, and/or between rather than within-subject design without a baseline measure. Note that, statistical analyses that simultaneously take multiple factors into consideration might be more appropriate than *t* test for study comparison.

In sum, the effect of alcohol consumption on stimulus-driven inhibition was less robust as one might have expected. In fact, nearly half of the studies that used the stop-signal task failed to identify a significant main effect of alcohol (see Table S1a here, and Table 5 in Bartholow et al., 2018). By contrast, studies used the cued go/no-go task (Marczinski, Abroms, Van Selst, & Fillmore, 2005) all confirmed the acute alcohol effect (Bartholow et al., 2018). A potential reason is that the prepotency/urgency of stopping is increased by invalid go cues in the cued go/no-go (Bartholow et al., 2018). Furthermore, alcohol may influence inhibitory control only during the decreasing limb of BAC (Bartholow et al., 2018), which helps explain the less apparent effect when the whole BAC curve was considered. As a next step, researchers can consider adding (in)valid cues into the stop-signal task and investigate why alcohol influences inhibition as a function of the BAC curve.

In addition, we found an interaction between Sex and the Pharmacological effect of alcohol use on SSRT. That is, females who drank alcohol had significantly shorter SSRT than females in the other two (non-alcohol) groups, and males who did not drink alcohol (placebo and control) had shorter SSRT than females. However, both effects were likely due to the prolonged SSRT of females in the placebo group (Fig. 3a), which was not due to strategical slowing-down of go RT as go RT was comparable with females in the other two groups (Fig. 3b).

### Acute alcohol use and intentional inhibition

Our most important finding is a pharmacological effect of alcohol on intentional inhibition. Participants who received alcohol stopped less often compared with participants in the placebo and control groups. This is in line with the assumption that alcohol may cause an intentional inhibition impairment, which contributes to increased drinking or loss of control over alcohol-seeking behavior (Field et al., 2010). This finding might pertain to attention narrowing and/or delay aversion. First of all, alcohol is hypothesized to induce a narrowing of the attentional focus, such that dominant cues become the center of attention and peripheral cues are ignored (Steele & Josephs, 1990). In the current context, tracking bottles is the primary assignment; thinking about disengagement and the corresponding reward is of secondary concern. Accordingly, after alcohol intake, participants might be more focused on the things at hand and prefer to continue tracking. Alternatively, delay aversion might be enhanced after alcohol intake. In the chasing bottles task, to continue tracking yielded greater immediate reward, whereas disengagement was associated with future reward. Thus, choosing not to stop to some extent reflects a myopia for the future, which is associated with alcohol intake (Reynolds et al., 2006). The pharmacological effect of alcohol in the survival analysis was replicated by a traditional ANOVA^6^ of the whether component (see details in supplementary materials **S3**). In contrast, the when component-related ANOVA indicated no effect of acute alcohol use. Results on the when component coincide with our prior work on the long-term effect of alcohol use (Liu et al., 2020). The divergent findings on different components of intentional inhibition is supported by separable underlying neuromechanisms (see Zapparoli et al., 2018), and emphasized alcohol’s unique effect on the whether component. In terms of the alcohol expectancy effect, it seems that participants adopted a compensatory strategy to counteract perceived disruptive effects by not stopping during the first 6 s (Marczinski & Fillmore, 2005). The remaining 14 s showed an opposite, pharmacologically driven reduction in intentional inhibition.

Note that at present we cannot distinguish beyond doubt whether alcohol affects the efficacy of intentional inhibition itself, or instead leads to failures to trigger the inhibitory process. While the analogous literature on stop tasks appears to suggest that alcohol affects the efficacy of stopping, it cannot be excluded there as well that alcohol results in trigger failures. Trigger failures may be closely related to the process of inhibition itself, but may also represent a process somewhat more remote from inhibition (e.g., a lack of attentional detection of the stop-signal, Matzke, Hughes, Badcock, Michie, & Heathcote, 2017; Matzke, Love, & Heathcote, 2017). The literature is only beginning to distinguish and unravel the two. At this stage we acknowledge this potential cavity, leaving open whether alcohol affects the inhibitory process itself or the failure to trigger it.

### The salience of alcohol-related stimuli

An important neuroadaptation in addiction is that the brain’s wanting system becomes hypersensitive (“sensitized”) to drugs and drug-associated stimuli (Robinson & Berridge, 1993). Stimulus type (alcoholic vs. non-alcoholic words) did not affect stimulus-driven inhibition in our sample of non-dependents, similar to what Zack et al. (2011) found. Confrontation with alcohol-associated stimuli might induce response inhibition deficits in substance abusers, and more so when dependence progresses (Robinson & Berridge, 2001). In the chasing bottles task, contrary to our hypothesis, those who drank alcohol had a higher stopping probability when tracking alcohol-related than soft-drink bottles. This counterintuitive pattern matches findings from others (Adams, Ataya, Attwood, & Munafò, 2013; Monk, Qureshi, Pennington, & Hamlin, 2017). A possible explanation is that a general difficulty in inhibiting appetitive (e.g., alcohol, water) versus non-appetitive stimuli (e.g., washing liquid, Monk et al., 2017) is likely to be formed once a motivational state was activated (Wadhwa, Shiv, & Nowlis, 2008). Alternatively, some participants may successfully teach themselves to treat alcohol-related stimuli as a stop-signal after drinking (cf. Fishbach & Shah, 2006). In a broader picture, the incentive-sensitization theory was only partially confirmed by Jones et al. (2018) recent meta-analysis. They found that the exacerbated impairment caused by appetitive cues disappeared after correcting for publication bias. Future studies might consider inducing a stronger effect by creating a multi-sensory substance-related context (visual, olfactory and locomotor, Field & Jones, 2017).

### Stimulus-driven inhibition versus intentional inhibition

A possible explanation of the divergent findings between different tasks is that intentional inhibition and stimulus-driven inhibition are considered to be fundamentally distinct (Ridderinkhof, van den Wildenberg, & Brass, 2014). This raises the importance of introducing intentional inhibition to the addiction-related field as its deficits might underlie the entrenched pattern of drinking. Contemplating on these findings gives rise to an interesting speculation, such that stimulus-driven inhibition and intentional inhibition displayed vulnerability to alcohol at different stages of a drinking episode. Intentional inhibition is likely to be influenced by a small to moderate amount of alcohol, which promotes further consumption. Afterward, when the accumulated consumption reaches a threshold, stimulus-driven inhibition is likely to be impaired, reflected by impulsive behavior. This hypothesis needs further testing.

### Limitations and future directions

Some limitations should be mentioned. First, the BrAC levels varied between the tasks and were relatively lower than expected. Using an alcohol clamping method that minimizes the variability might be considered for future research (Ramchandani et al., 2006). Second, in the chasing bottles task, a longer tracking period was not always associated with more immediate rewards. This may be criticized as it did not mimic the ever-increasing pleasure acquired from continued drinking in reality. However, if the immediate reward kept increasing during that 20 s, the premium response (i.e. to disengage tracking just before the 20-s window elapsed to maximize their total reward) would be very likely to be executed, which discourages intentional inhibition. Such criticism does not apply to non-stopped trials as it indeed produced immediate reward and thus was closer to reality. This also helps explain the effect of alcohol particularly on the whether component. A better balance between free will and ecological validity was required for the task. Third, incorporating incentive feedback in the chasing bottles task might have tapped into other cognitive processes such as strategy learning. This rewarding system was designed on purpose, as one feature of volition is reason-responsive (i.e. all (non-)actions have a reason, Haggard, 2018). Unfortunately, it introduced some adverse influence in addition to the benefits. Fourth, the 20-s time-window for participants to decide to stop tracking was rather arbitrary. In other words, if a longer decision period was allowed, such as 60 s, the intentional stopping probabilities might have been different. This can be argued against, as alcohol consumption consistently decreased the stopping rate at any time point within that 20 s (i.e., survival curves representing the alcohol condition were consistently above the other two conditions), and in reality, one hardly hesitated/struggled for even longer time before deciding to accept/reject the next beer. Fifth, calories contained in the drinks differed between groups (approx. alcohol group: 400 kcal, placebo group: 200 kcal, control group: 0). Although sugar levels influence sports performance (Campbell, Prince, Braun, Applegate, & Casazza, 2008), its role in response inhibition is rather unclear. Still, future studies might refine the procedure of placebo design to balance the amount of calories between groups (e.g., administer orange juice rather than water to the control group, see Stoner et al., 2008).

## Conclusion

In the current study, we investigated the acute effect of alcohol on two forms of response inhibition: stimulus-driven inhibition and intentional inhibition. Alcohol intake did not systematically affect stimulus-driven inhibition. It shortened females’ SSRT compared to placebo and control. Males in the placebo and control groups had shorter SSRT than females. Intentional inhibition, as tested with the chasing bottles task, was negatively influenced by alcohol intake. Participants who drank alcohol were less likely to intentionally stop their bottle-tracking behavior. In sum, stimulus-driven inhibition and intentional inhibition represented two types of response inhibition; the importance of intentional inhibition in the development and maintenance of addiction should be considered.

## Electronic supplementary material

Below is the link to the electronic supplementary material.

Supplementary file1 (DOCX 887 kb)

## Footnotes

1 Only some specific measures of inhibitory control (e.g., Stroop conflict) but not others (e.g., response inhibition) are associated with self-reported impulsivity (Enticott, Ogloff, & Bradshaw, 2006).

2 The two mid-of-task measurements.

3 The reparametrized factor consume alcohol and expectancy of alcohol was used in the model, as the original factor drink condition (alcohol/placebo/control) violated the proportional hazard assumption of the Cox regression that was used in the chasing bottles task and we would like to keep it consistent between tasks. Mathematically, results from reparametrized models are equivalent, and descriptive or predictive powers of both models are the same. The reparametrized model provided qualified estimations of the ‘upper-level’ variables that are behind this grouping (i.e., the Pharmacological and Expectancy effects of alcohol).

4 Both predictors violated the proportional hazard assumption (i.e., the effect was not consistent over time. see Fig. S2a, S2b) and this was accounted for by stratification (Schemper, 1992).

5 The *χ*^2^ and *p* value refer to the overall contribution of a certain variable to the model. In contrast, the hazard ratios and CI represent results when comparing two specific scenarios at fixed values of other predictors (here compared to the reference level). Reference levels: consume alcohol (no), bottle category (soft drink), and task sequence (stop-signal task as the first task).

6 Traditional ANOVAs were performed for confirmation.
