# Supplementary material for: Moderate acute alcohol use impairs intentional inhibition rather than stimulus-driven inhibition
Source: Psychol Res. 2020 May 19;85(4):1449–61. doi: 10.1007/s00426-020-01353-w (PMC8211579; doi:10.1007/s00426-020-01353-w)
Supplement: Supplementary file 1 — Supplementary file1 (DOCX 887 kb) [file 426_2020_1353_MOESM1_ESM.docx]

**Article Title**

Moderate acute alcohol use impairs intentional inhibition rather than stimulus-driven inhibition

**Journal Name**

Psychological Research

**Authors Names**

Yang Liu*, Raoul P.P.P. Grasman, Reinout W. Wiers, K. Richard Ridderinkhof, Wery P.M. van den Wildenberg

*corresponding author

**Affiliations**

^a^Department of Psychology, University of Amsterdam, Amsterdam, the Netherlands

^b^Addiction, Development, and Psychopathology (ADAPT) Lab, Department of Psychology, University of

Amsterdam, Amsterdam, the Netherlands

^c^Amsterdam Brain & Cognition (ABC), University of Amsterdam, Amsterdam, the Netherlands

**Email of YL:** [yliux@outlook.com](mailto:yliux@outlook.com)

**Contents**

**S1. Questionnaires:** Introduction, Analyses, and Results

**S2. Chasing Bottles task:** SSRT and personalities in predicting stopping probability

**S3: Chasing Bottles task:** analytical approach and results with ANVOA

**Tables**

**Table S1a** Overview of studies on acute alcohol use and SST

**Table S1b** Comparison of studies had positive findings vs. those had negative findings

**Table S2** Stimuli used in the stop-signal task

**Table S3a–3c** Stop-signal task: Repeated-measures ANOVA results of SSRT, go RT and stop rate for **Words**

**Table S4a–3c** Stop-signal task: ANOVA results of SSRT, go RT and stop rate for **Non-Words**

**Figures**

**Fig. S1**. Bottles used in the Chasing Bottle task

**Fig. S2a**. The expectancy effect of alcohol on the hazard rate over time

**Fig. S2b.** The effect of sex on the hazard rate over time

**Fig. S3**. Breath Alcohol Concentration Readings at each time point

**Fig. S4a & S4b.** Simulant and Sedative subscale of Brief Biphasic Alcohol Effects for alcohol group and placebo group at different time points

**Short Clips**

**References**

**S1. Questionnaires**

*Alcohol use disorder identification test* (AUDIT, Saunders et al., 1993): The AUDIT is a 10-item survey used as a screening instrument for excessive or hazardous alcohol use. A total score of 8 is a reasonable cut-off for a variety of adverse outcomes (Conigrave, Hall, & Saunders, 1995). It has good reliability with a median Cronbach’s alpha of 0.80 in a review (Reinert & Allen, 2002).

*Core alcohol and drug survey* (CORE, Presley, 1993): The examines the use, scope, and consequences of alcohol and other drugs in college settings (Presley, 1993). Respondents were asked to indicate how often within the last month they had used each of the 11 specific types of drugs (alcohol, tobacco, marijuana, cocaine, amphetamines, sedatives, hallucinogens, opiates, inhalants, designer drugs, and steroids).

*Desire for Alcohol Questionnaire* (DAQ, Love, James, & Willner, 1998): The DAQ is developed to measure three aspects of craving: (a) strong desires/intentions to drink (DAQ-crave), (b) negative reinforcement (DAQ-negative), and (c) positive reinforcement and ability to control drinking (DAQ-positive) (Kramer et al., 2010). The 14-item questionnaire uses a 7-point Likert scale, ranging from “strongly disagree” to “strongly agree”. Reliability of the DAQ-total score and subscale scores were found to be adequate with Cronbach’s α of .70, .70, .76 and .86 respectively (Courtney et al., 2013).

*Positive and Negative Affect Scale* (PANAS, Watson, Clark, & Tellegen, 1988): The PANAS is used to provide measures of positive and negative affect. The 20 items describe either a positive (e.g., interested) or a negative (e.g., distressed) feeling or mood state. Subjects are asked to indicate to what extent these words describe how they feel at the present moment, or have felt over the past week. The PANAS uses a 5-point Likert scale ranging from “very slightly” to “very much”. The Cronbach’s alpha reliabilities for both scales are high, generally ranging from .83 to .90 for Positive Affect, and from .85 to .90 for Negative Affect (Watson & Clark, 1999).

*Self-Rating of the Effects of Alcohol* (SRE, Schuckit et al., 1997): The individual level of response to alcohol can be determined with the 12-item SRE. Subjects are asked to indicate how many alcoholic beverages they would need to elicit one of four possible effects on three different moments in time (i.e. the first five times they ever drank: SRE-5; 3 months of drinking once a month: SRE-3; the period of heaviest drinking: SRE-H, Schuckit, et al., 1997). The higher the SRE score, the lower the sensitivity to alcohol (Piasecki et al., 2012). SRE has good reliabilities in both self-reported and interview administrations (Ray, Hart, & Chin, 2011).

*Rutgers Alcohol Problem Index* (RAPI, White & Labouvie, 1989): The RAPI is a 23-item screening tool used to assess alcohol-related problems. It uses a 4-point Likert scale: never, 1-2 times, 3-5 times, and more than 5 times. Subjects are asked to indicate how many times they experienced each statement, e.g., “Not able to do your homework or study for a test”. The coded numbers (0-3) were added together across items to form a scale ranging from 0 to 69. This scale has a reliability of .92 and a 3-year stability coefficient of .40 for the total sample (White & Labouvie, 1989).

*Dickman’s impulsivity inventory* (DII, Dickman, 1990): The DII includes two subscales to measure dysfunctional and functional impulsivity. The 23 items are in a yes/no format. Eleven items focus on functional impulsivity (e.g., “People have admired me because I can choose quickly”). Twelve other items tap dysfunctional impulsivity (e.g., “I often say and do things without considering the consequences”). Cronbach’s alpha coefficient for the Dutch version was 0.84 for the dysfunctional dimension and 0.76 for the functional dimension (Claes, Vertommen, & Braspenning, 2000).

*Sensitivity to punishment and sensitivity to reward questionnaire* (SPSRQ, Torrubia et al., 2001): The SPSRQ is a 48-item yes/no response questionnaire developed to assess the Behavioral Inhibition System (BIS) and the Behavioral Activation System (BAS). The Sensitivity to Punishment scale (SP) measures behavioral inhibition under specific conditions of threat or punishment; and the Sensitivity to Reward scale (SR) reflects approach behavior to specific conditioned and unconditioned rewards, notably money, social status and sexual partners (Dawe & Loxton, 2004). The reliability of the SR and SP subscales in this study was good (i.e. Cronbach’s alpha of 0.70, and 0.83, respectively).

*Brief Biphasic Alcohol Effects Scale* (B-BAES, Rueger et al., 2009): The 6-item B-BAES is a measure of alcohol’s acute stimulant and sedative effects. The stimulation subscale consists of the adjectives energized, excited and up, while the sedative subscales make use of the words sedated, slow thoughts and sluggish. Both subscales use an 11-point Likert scale ranging from 0 (“not at all”) to 10 (“extremely”). Subjects in the alcohol and placebo condition filled it out each time a breath sample was taken except for the baseline.

**Analyses**

For all these questionnaires (i.e., DAQ, PANAS, SRE, PAPI, DII, SPSRQ), a one-way ANOVA was carried out with Drink Condition (Alcohol/Placebo/Control) as the independent variable. For the evaluation of bottles (i.e., those used in the Chasing Bottle task), three parallel one-way MANOVAs were carried out for pleasant, arousal and dominance separately. For each MANOVA, evaluations of 10 different bottles were the dependent variables and Drink Condition was the independent variable.

**Results**

Comparison between groups on questionnaires (N = 106)

| Variables | Alcohol | Placebo | Control | *F* | *P* |
| --- | --- | --- | --- | --- | --- |
|  | (N = 33) | (N = 36) | (N = 37) |  |  |
|  | *M (SD)* | *M (SD)* | *M (SD)* |  |  |
| DAQ |  |  |  |  |  |
| Crave | 13.67(4.31) | 13.22(5.08) | 13.28(3.93) | 0.10 | 0.91 |
| Negative | 10.73(5.18) | 9.36(3.35) | 8.75(2.97) | 2.29 | 0.11 |
| Positive | 20.18(2.72) | 19.58(2.69) | 20.22(3.00) | 0.58 | 0.56 |
|  |  |  |  |  |  |
| PANAS |  |  |  |  |  |
| Positive | 32.79(5.95) | 33.22(6.22) | 32.83(5.79) | 0.06 | 0.95 |
| Negative | 16.94(4.74) | 16.61(5.11) | 16.03(5.08) | 0.30 | 0.74 |
|  |  |  |  |  |  |
| SRE |  |  |  |  |  |
| SRE-5 | 4.27(1.58) | 4.34(1.27) | 4.28(1.61) | 0.02 | 0.98 |
| SRE-3 | 6.16(2.01) | 6.66(2.01) | 6.62(2.55) | 0.55 | 0.58 |
| SRE-H | 8.11(2.72) | 7.90(2.80) | 7.90(3.35) | 0.06 | 0.95 |
|  |  |  |  |  |  |
| RAPI | 9.24(6.56） | 7.20(4.12） | 8.27(5.84) | 1.20 | 0.306 |
|  |  |  |  |  |  |
| DII |  |  |  |  |  |
| Functional | 7.64(2.06) | 7.50(2.50) | 7.70(2.58) | 0.04 | 0.96 |
| Dysfunctional | 2.85(2.70) | 2.39(2.03) | 3.11(2.37) | 0.86 | 0.43 |
|  |  |  |  |  |  |
| SPSRQ |  |  |  |  |  |
| Punishment | 9.31(5.44) | 7.72(4.55) | 9.27(4.50) | 1.25 | 0.29 |
| Reward | 12.78(4.20) | 12.75(3.14) | 14.22(3.42) | 1.96 | 0.15 |

*Note.* DAQ: Desire for Alcohol Questionnaire, PANAS: Positive and Negative Affect Scale,

SRE: Self-Rating of the Effects of Alcohol, RAPI: Rutgers Alcohol Problem Index, DII:

Dickman’s impulsivity inventory, SPSRQ: Sensitivity to punishment and sensitivity to

reward questionnaire.

The above table listed the descriptive and statistical data for comparison between the three groups. There were no significant differences in all these questionnaires and their subscales between the three groups (all *p* ≥ 0.15).

The MANOVA revealed non-significant associations between the pleasantness of bottles and Drink Condition (*F* (4, 214) = 0.70, *Wilk's Λ* = 0.97, *p* = 0.59). Similarly, there were non-significant associations between the arousal of bottles and Drink Condition (*F* (4, 214) = 1.61, *Wilk's Λ* = 0.96, *p* = 0.33) and dominance values of bottles and Drink Condition (*F* (4, 214) = 1.76, *Wilk's Λ* = 0.94, *P* = 0.14). These findings together indicated that the three groups have similar evaluations of alcohol bottles and soft drink bottles in terms of pleasantness, arousal, and dominance.

**S2. Chasing Bottles task: SSRT and personalities in predicting stopping probability**

SSRTs to alcohol-related words did not predict stopping probability for alcoholic bottles (*HR* = 1.00, *p* = 0.43, 95% CI [0.999, 1.00]). Similarly, SSRTs to neutral words did not predict stopping probability for soft drink bottles (*HR* = 1.00, *p* = 0.46, 95% CI [0.997, 1.00]). This indicated that stimulus-driven inhibition, as assessed by SST, and intentional inhibition, assessed by the Chasing Bottle task, represented different aspects of response inhibition. In addition, none of the following personalities predicted stopping probability, functional impulsivity (*HR* = 1.04, *p* = 0.08, 95% CI [1.00, 1.08]), dysfunctional impulsivity (*HR* = 1.02, *p* = 0.31, 95% CI [0.98, 1.07]), and reward sensitivity (*HR* = 0.98, *p* = 0.06, 95% CI [0.96, 1.00]). This indicated that inhibition measured by the Chasing Bottles task was unrelated to trait impulsivity measured by questionnaires; variances of stopping probability cannot be explained by the variance of reward sensitivity.

**S3: Chasing Bottles task:** analytical approach and results with ANVOA

For the Chasing Bottles task, two dependent variables (e.g., stop rate and stop latency) were calculated separately for different bottle categories. For stop latency, trials without a stop within the 20s time window were discarded. Two four-way repeated-measures ANOVAs (2(Pharmacological effect) × 2(Expectancy effect) × 2(Sex) × 2(Bottle Category)) were performed while controlling for task sequence. As to the stop rate, only the Pharmacological effect of alcohol was significant (*F*(1, 94) = 4.34, *p* = 0.04). Participants who drank alcohol stopped less often (*M* = 0.6, *SD* = 0.25) than those did not drink alcohol (*M* = 0.73, *SD* = 0.20). The effect of other factors and their interactions were not significant, including Task Sequence (F(1, 94) = 0.46, p= 0.50) , Sex (*F*(1, 94) = 0.001, *p* = 0.97), Bottle Category (*F*(1, 94) = 0.17, *p* = 0.90), Alcohol Expectancy (F(1, 94) = 0.31, p = 0.58), Bottle Category × Sex (*F*(1, 94) = 0.001, *p* = 0.97), Alcohol Expectancy × Sex (*F*(1, 94) = 0.43, p = 0.51), Alcohol Pharmacology × Sex (*F*(1, 94) = 1.25, p = 0.27), Bottle Category × Alcohol Expectancy (*F*(1, 94) = 1.95, *p* = 0.17), Bottle Category × Alcohol Pharmacology (*F*(1, 94) = 1.73, *p* = 0.19), Bottle Category × Alcohol Expectancy × Sex (*F*(1, 94) = 0.15, *p* = 0.69), Bottle Category × Alcohol Pharmacology × Sex (*F*(1, 94) = 0.16, *p* = 0.69). As to the stop latency, the effect of all predictors were not significant, including Task Sequence (F(1, 94) = 0.46, p = 0.50), Sex (*F*(1, 94) = 0.001, *p* = 0.97), Bottle Category (*F*(1, 94) = 0.001, *p* = 0.97), Alcohol Pharmacology (*F*(1, 94) = 0.05, *p* = 0.83), Alcohol Expectancy (*F*(1, 94) = 0.04, *p* = 0.84), Bottle Category × Sex (*F*(1, 94) = 0.73, *p* = 0.40), Alcohol Expectancy × Sex (*F*(1, 94) = 0.02, *p* = 0.88), Alcohol Pharmacology × Sex (*F*(1, 94) = 0.01, *p* = 0.93), Bottle Category × Alcohol Expectancy (*F*(1, 94) = 0.95, *p* = 0.33), Bottle Category × Alcohol Pharmacology (*F*(1, 94) = 1.33, *p* = 0.25), Bottle Category × Alcohol Expectancy × Sex (*F*(1, 94) = 2.81, *p* = 0.10), Bottle Category × Alcohol Pharmacology × Sex (*F*(1, 94) = 1.44, *p* = 0.23).

**Table S1a** Overview of studies on acute alcohol use and stop-signal task

| **Study** | **Stop signal modality** | **Male %** | **Drinks per week** | **Alcohol to placebo comparison** | **Pre-drink/ baseline condition** | **N** | **Number of trials** | **Ratio of stop** | **Alcohol dose** | **SSRT calculation** | **Main findings** | **Effect size** | **Positive finding?^§^** |
| --- | --- | --- | --- | --- | --- | --- | --- | --- | --- | --- | --- | --- | --- |
| [Bartholow et al. (2018)](http://psycnet.apa.org/record/2018-25407-004) | Visual | 50% | 7.5 | Between (alcohol/placebo/control) | Yes | 216 | 160 | 25% | 0.80 g/kg (men), 0.72 g/kg (women) | Median | The stop-signal performance was impaired by alcohol relative to placebo and control only on the decreasing limb of BrAC. | η_p_^2^ = 0.07 | Partially |
| [Campbell et al. (2017)](https://www.sciencedirect.com/science/article/pii/S0376871617304635) | Visual | 37.50% | Audit < 16, at least 6 binge occasions for the past year. | Within (alcohol/placebo) | Yes | 40 | 288 | 25% | 0.8 g/kg | Integration | Time (pre-post drink)× condition (alcohol/placebo) was significant. post-hoc test was not reported. | η_p_^2^ = 0.13 | Yes |
| [Caswell et al. (2013)](https://link.springer.com/article/10.1007/s00213-013-3079-8) | Visual | 50% | 19.8 | Between (placebo/low dose/high dose) | No | 48 | 120 | 25% | 0.4g/kg or 0.8 g/kg | Integration | Only high dose induced longer SSRT than placebo. | η_p_^2^ = 0.16 | Partially |
| [de Wit et al. (2000)](https://www.ncbi.nlm.nih.gov/pubmed/10959541) | Auditory | 70% | 5 | Within | Yes | 17 | 64 | 25% | 0.2 g/kg, 0.4 g/kg, 0.8 g/kg | NA, SSRT was calculated from the last block, 64 trials. | The 0.4 g/kg and 0.8 g/kg dose prolonged SSRT compared to placebo. | η_p_^2^ = 0.26 | Yes |
| [Dougherty et al. (2008)](https://www.sciencedirect.com/science/article/pii/S0376871608000707?via%3Dihub) | Visual | 50% | 10(male), 6(female) | Within (placebo and four different doses of alcohol) | Yes (tested -0.5h, 0.25h, 1h, 2h after drinking) | 30 | NA | 25% | 0.2, 0.4, 0.6, 0.8 g/kg | NA, stop rate is the main dependent variable. | Only a main effect of time was found (impulsive response kept increasing), regardless of alcohol dose. | NA | No |
| [Dougherty et al. (2015)](https://www.ncbi.nlm.nih.gov/pmc/articles/PMC4388789/pdf/nihms659922.pdf) | Visual | 60% | 19(male), 22(female) | Between (placebo/alcohol) | Yes | 179 | NA | 25% | 0.3 k/kg × three times separated by one hour each. | NA, stop rate is the main dependent variable. | Compared with placebo, alcohol intake increased the rate of failed inhibition. | NA | Yes |
| [Easdon & Vogel-Sprott, (2000)](https://doi.org/10.1037/1064-1297.8.3.387) | Auditory | 100% | 10 | Between (alcohol/placebo) | Yes | 16 | 176 | 28.4% | 0.62 g/kg | NA | Compared to baseline, alcohol caused more failed inhibition, which was not the case for placebo. | η_p_^2^ = 0.46 | Yes |
| [Fillmore & Blackburn, (2002)](https://www.jsad.com/doi/abs/10.15288/jsa.2002.63.237) | Auditory | 81.25% | 7 | Between (control/alcohol/placebo) | Yes | 48 | 176 | 28.4% | 0.65 g/kg | NA | People drank alcohol failed more inhibition compared with placebo and control. | η_p_^2^ = 0.19 | Yes |
| Fillmore & Vogel-Sprott, ([1999](https://www.jsad.com/doi/10.15288/jsa.2000.61.239), [2000](https://www.jsad.com/doi/10.15288/jsa.2000.61.239)) | Auditory | 100% | 7 | Between (alcohol/placebo) | Yes | 14 | 176 | 27% | 0.62 g/kg | NA | Compared to baseline, alcohol caused more failed inhibition, which was not the case for placebo. | NA | Yes |
| [Gan et al. (2014)](https://www.sciencedirect.com/science/article/pii/S0006322314000158) | Visual | 74% | 10 | Within (alcohol/placebo) | No | 42 | 320 | 20% | 0.6 g/kg | Integration | Alcohol significantly prolonged SSRT. | Cohen's d = 0.63 | Yes |
| [Guillot et al. (2010)](https://www.ncbi.nlm.nih.gov/pmc/articles/PMC3968820/) | Visual | 44% | About 10.5 | Between (placebo/alcohol) | No | 141 | 100 | 80% | Target BAC: 0.00%, 0.05%, 0.075% and 0.10% | NA, stop rate is the main dependent variable. | No main effect of alcohol, nor its interaction with gender was significant. | NA | No |
| [Kareken et al. (2013)*](https://doi.org/10.1007/s00213-013-3038-4) | Visual | 61% | 13.9 | Within (placebo/alcohol) | No | 18 | 360 | 66.7% | BrAC of 0.06% | Integration | Alcohol infusion produced longer SSRT than placebo saline infusion. | NA | Yes |
| Loeber and Duka (2009)^a^ | Auditory | 53% | 24 | Between (placebo/alcohol) | Yes | 36 | 320 | 25% | 0.8 g/kg | Median | Time × group interaction was found. Post-hoc test found alcohol group had lengthened SSRT compared to baseline. No clear difference between alcohol and placebo at T2 was reported. | Time x Group Interaction η_p_^2^ = 0.13; | Partially |
| Loeber and Duka (2009)^b^ | Auditory | 50% | 23.2 | Between (placebo/alcohol) | Yes | 32 | 320 | 25% | 0.8 g/kg | Median | Only marginal interaction between time and group was found. The post-hoc test revealed that the alcohol group had longer SSRT at T2 compared to baseline. And no clear difference between alcohol and placebo group at T2 was reported. | Time x Group Interaction η_p_^2^ = 0.11 | No |
| Loeber and Duka (2009)^c^ | Auditory | 50% | 24.5 | Between (placebo/alcohol) | Yes | 32 | 320 | 25% | 0.8 g/kg | Median | Alcohol group had longer SSRT compared with placebo after drink, but no difference at baseline. | Time x Group Interaction η_p_^2^ = 0.52; Post-hoc alcohol vs. placebo post drinking: Cohen's d = 1.40 | Yes |
| [McCarthy et al. (2012)](https://onlinelibrary.wiley.com/doi/full/10.1111/j.1360-0443.2012.03974.x) | Visual | 55% | NA | Within alcohol (increasing & decreasing limb) vs control | No | 29 | NA | NA | 0.72 g/kg for men, 0.65 g/kg for women | NA | Alcohol session produced marginally longer SSRT (p=0.052). | η_p_^2^ = 0.12 | No |
| [Mulvihill et al. (1997)](https://www.jsad.com/doi/abs/10.15288/jsa.1997.58.600) | Auditory | 50% | 5.5 | Between (alcohol/placebo/control) | Yes | 48 | 176 | 27% | 0.62 g/kg for men, 0.54 g/kg for women |  | Compared to baseline, alcohol caused more failed inhibition, which was not observed for the placebo and control group. | η_p_^2^ = 0.22 | Yes |
| [Nikolaou et al. (2013)](https://journals.plos.org/plosone/article?id=10.1371/journal.pone.0076649) | Visual | 50% | 26 | Between (placebo, low dose, high dose) | Yes | 42 | 120 | 25% | 0.4 g/kg or 0.8 g/kg | Mean | No difference at baseline, however, both alcohol groups had longer SSRT compared with placebo. | Time x Group Interaction: η_p_^2^ = 0.156, High dose vs placebo: Cohen's d = 0.93 | Yes |
| [Peacock et al. (2015)](https://onlinelibrary.wiley.com/doi/full/10.1111/acer.12680) | Visual | 100% | 7.4 | Within (placebo/alcohol/alcohol mixed with 500ml energy drink/alcohol mixed with 750ml energy drink) | No | 19 | 48 per session, three sessions (0.05% ascending BrAC, ~0.08% peak BrAC, and ~0.05% descending BrAC) | 25% | Target BAC: 0.05% and 0.08% after first and second administration, respectively. | Mean | No main effect of condition, time and their interaction. Only when BrAC was 0.08%, the alcohol mixed with 750ml energy drinks condition had longer SSRT than the alcohol condition. | Cohen's d = 0.37 | No |
| [Plawecki et al. (2018)](https://www.sciencedirect.com/science/article/pii/S074183291730808X) | Visual | 50% | 14.5 | Within (alcohol/placebo) | Yes | 49 | 180 | 33% | BrAC 60 mg/dL | NA | Alcohol significantly reduced P3 amplitude in the slow SSD compared to the fast SSD group, but significantly increased P3 latency in the fast SSD compared to the slow SSD group. | NA | NA |
| [Reynolds et al. (2006)](https://www.sciencedirect.com/science/article/pii/S0091305706000219) | Auditory | 46% | 6.6 | Within (placebo/ low dose/high dose) | No | 24 | NA | 25% | 0.4g/kg or 0.8 g/kg | Median | Both low and high dose alcohol consumption lengthened SSRT compared to placebo. | η_p_^2^ = 0.30 | Yes |
| [Spinola et al. (2017)](https://www.sciencedirect.com/science/article/pii/S0741832916301586?via%3Dihub) | Auditory | 48% | NA (moderate to heavy drinkers) | Within (control/placebo/alcohol) | Yes | 75 | 192 | 25% | 0.65g/kg | NA | No beverage condition or beverage condition × time interaction effects on inhibition were found. | beverage group × time: η_p_^2^ = 0.04, beverage group: η_p_^2^ = 0.02 | No |

*Note.* BrAC: Breath Alcohol Concentration, NA: not available, *descriptive data shown in this table were from the family history negative group only, ^§^In total, 7 studies found null effect of alcohol, 11 studies found impairing effect of alcohol on stop signal performance, 3 studies partially confirmed the impairing effect.

**Table S1b** Comparison of studies had positive findings vs. those had negative findings

| Variables | Positive findings | Negative findings | *t* | *p* |
| --- | --- | --- | --- | --- |
|  | (n = 16^1^) | (n = 12^1^) |  |  |
| Male percentage | 62.23 (18.92) | 55.08 (15.64) | -1.06 | 0.30 |
| Units of alcohol/week | 15.71 (13.28) | 10.85 (5.88) | -1.08 | 0.29 |
| Sample size | 40.69 (39.06) | 51.83 (44.28) | 0.71 | 0.49 |
| Number of trials | 190.77 (99.81) | 134.86 (93.77) | -1.22 | 0.24 |
| Stop signal probability | 27.97 (10.50) | 35.00 (22.25) | 0.98 | 0.35 |
| Stop signal (auditory/visual) | 9/7 | 3/9 | *χ^2^=* 2.73 | 0.10 |
| Study design (between/within-subject) | 9/7 | 4/8 | *χ^2^*= 1.45 | 0.23 |
| Baseline measure (yes/no) | 11/5 | 7/5 | *χ^2^*= 0.32 | 0.57 |
| Alcohol dose administered (high/medium/low/super low)^2^ | 0/2/7/7 | 2/2/3/4 | *χ^2^*= 3.62 | 0.31 |

*Note.* Mean (Standard Deviation)

^1^For studies administered a different amount of alcohol, we treated them as multiple cases;

^2^high: more than 0.65g/kg, medium: 0.65g/kg to no more than 0.4g/kg, low: 0.4g/kg to more

than 0.2g/kg, super low: no more than 0.2g/kg.

**Table S2** Stimuli used in the stop-signal task

| **Neutral words**  **(n = 40)** | **Alcohol-related words**  **(n = 40)** | **Corresponding non-words**  **(n = 80)** | |
| --- | --- | --- | --- |
| prothesen | alcoholisch | priseson | alhevadich |
| huig | fust | syoi | fult |
| promo | baco | prami | bazo |
| verhuiswagen | kroegentocht | verleukwaben | krielenbosch |
| eiffeltoren | bacardi | eiffilbaken | banarvo |
| oplezen | bavaria | opkezen | bamalia |
| wegvaren | brandewijn | weglarpen | bravolijf |
| zeebaars | jenever | zeemijrs | jegevol |
| vlaktes | brouwen | vlintis | breizen |
| voltage | likeur | vokleume | libeut |
| zuidwest | heineken | zijdpast | hoevekel |
| taille | pils | tauti | pirs |
| zakmes | merlot | zakfos | murlos |
| kleven | tappen | klabon | tappin |
| decennia | slijterij | deseunijke | sleutekij |
| vuilnisbakken | aangeschoten | veuperlaazen | aanbeschalen |
| berechten | dronkenschap | bewochten | droewenschip |
| kous | port | koum | pijrt |
| reizigers | brouwerij | reimigors | breikerij |
| musical | cocktail | mivicol | cochlirt |
| pijlen | zuipen | pijwen | zuimen |
| wolk | rum | wolg | rom |
| fraai | shot | froei | shab |
| puin | gin | peum | gon |
| lampen | tequila | lanven | teqoleu |
| staken | kater | stirgen | kijter |
| gelijke | cognac | gezeupe | cownam |
| heuvels | kroeg | hoekems | kroew |
| zonen | wodka | zoben | voplo |
| weglopen | uitgaan | wetlagen | uitgook |
| ogenblik | alcohol | ogelblot | alcijlijs |
| fabriek | borrel | faltreik | bozzim |
| eisen | proost | eiben | preuks |
| bomen | whisky | boben | whekijs |
| adres | drank | adlis | drand |
| been | bier | beol | biem |
| rol | wijn | rov | weun |
| namen | dronken | nonen | dromkin |
| koud | feest | kolp | fijst |
| slang | brak | sleung | braf |

**Table S3a**–**c** Stop-signal task: Repeated-measures ANOVA results of SSRT, go RT and stop rate for **Words**

| **S3a:** SSRT | | | | |
| --- | --- | --- | --- | --- |
| Predictor | df | *F* | *p* | *η*_p_^2^ |
| Task Sequence | 1/99 | .29 | .59 | .00 |
| Sex | 1/99 | .34 | .56 | .00 |
| Word Category | 1/99 | .10 | .75 | .00 |
| AlcoholExpect | 1/99 | 1.13 | .29 | .01 |
| AlcoholPharmacological | 1/99 | 1.02 | .31 | .01 |
| Word Category × Sex | 1/99 | .01 | .94 | .00 |
| AlcoholExpect × Sex | 1/99 | 1.60 | .21 | .02 |
| AlcoholPharmacological × Sex | 1/99 | 5.02 | .03* | .05 |
| Word Category × AlcoholExpect | 1/99 | .38 | .54 | .00 |
| Word Category × AlcoholPharmacological | 1/99 | 1.38 | .24 | .01 |
| Word Category × AlcoholExpect × Sex | 1/99 | .13 | .72 | .00 |
| Word Category × AlcoholPharmacological × Sex | 1/99 | .09 | .77 | .00 |

*Note.* * *p* < 0.05, AlcoholExpect: Expectancy effect of alcohol, AlcoholPharma: Pharmacological effect of alcohol

| **S3b:** go RT | | | | |
| --- | --- | --- | --- | --- |
| Predictor | df | *F* | *p* | *η*_p_^2^ |
| Task Sequence | 1/99 | .12 | .73 | .00 |
| Sex | 1/99 | 1.00 | .32 | .01 |
| Word Category | 1/99 | 1.03 | .31 | .01 |
| AlcoholExpect | 1/99 | .07 | .79 | .00 |
| AlcoholPharmacological | 1/99 | 1.47 | .23 | .01 |
| Word Category × Sex | 1/99 | .05 | .82 | .00 |
| AlcoholExpect × Sex | 1/99 | .06 | .81 | .00 |
| AlcoholPharmacological × Sex | 1/99 | 1.40 | .24 | .01 |
| Word Category × AlcoholExpect | 1/99 | .55 | .46 | .01 |
| Word Category × AlcoholPharmacological | 1/99 | .47 | .49 | .00 |
| Word Category × AlcoholExpect × Sex | 1/99 | .53 | .47 | .01 |
| Word Category × AlcoholPharmacological × Sex | 1/99 | .01 | .91 | .00 |

*Note.* AlcoholExpect: Expectancy effect of alcohol, AlcoholPharma: Pharmacological effect of alcohol

| **S3c:** Stop rate | | | | |
| --- | --- | --- | --- | --- |
| Predictor | df | *F* | *p* | *η*_p_^2^ |
| Task Sequence | 1/99 | .98 | .32 | .01 |
| Sex | 1/99 | .03 | .85 | .00 |
| Word Category | 1/99 | 1.45 | .23 | .01 |
| AlcoholExpect | 1/99 | .02 | .88 | .00 |
| AlcoholPharmacological | 1/99 | .01 | .93 | .00 |
| Word Category × Sex | 1/99 | .20 | .65 | .00 |
| AlcoholExpect × Sex | 1/99 | .42 | .52 | .00 |
| AlcoholPharmacological × Sex | 1/99 | .60 | .44 | .01 |
| Word Category × AlcoholExpect | 1/99 | .82 | .37 | .01 |
| Word Category × AlcoholPharmacological | 1/99 | .01 | .93 | .00 |
| Word Category × AlcoholExpect × Sex | 1/99 | .00 | .99 | .00 |
| Word Category × AlcoholPharmacological × Sex | 1/99 | .02 | .88 | .00 |

*Note.* AlcoholExpect: Expectancy effect of alcohol, AlcoholPharma: Pharmacological effect of alcohol

**Table S4a**–**c** Stop-signal task: ANOVA results of SSRT, go RT and stop rate for **Non-Words**

**S4a:** SSRT

| Predictor | df | *F* | *p* | *η*_p_^2^ |
| --- | --- | --- | --- | --- |
| Task sequence | 1 | 0.02 | 0.88 | 0 |
| Sex | 1 | 2.14 | 0.15 | 0.02 |
| AlcoholExpect | 1 | 0.14 | 0.71 | 0 |
| AlcoholPharma | 1 | 0.41 | 0.52 | 0 |
| AlcoholExpect × Sex | 1 | 0.12 | 0.73 | 0 |
| AlcoholPharma × Sex | 1 | 0.31 | 0.58 | 0 |

*Note.* AlcoholExpect: Expectancy effect of alcohol, AlcoholPharma: Pharmacological effect of alcohol

**S4b:** go RT

| Predictor | df | *F* | *p* | *η*_p_^2^ |
| --- | --- | --- | --- | --- |
| Task sequence | 1 | 0.02 | 0.89 | 0 |
| Sex | 1 | 0.05 | 0.83 | 0 |
| AlcoholExpect | 1 | 0.13 | 0.72 | 0 |
| AlcoholPharma | 1 | 0.2 | 0.66 | 0 |
| AlcoholExpect * Sex | 1 | 1.13 | 0.29 | 0.01 |
| AlcoholPharma * Sex | 1 | 0.92 | 0.34 | 0.01 |

*Note.* AlcoholExpect: Expectancy effect of alcohol, AlcoholPharma: Pharmacological effect of alcohol

**S4c:** Stop rate

| Predictor | df | *F* | *p* | *η*_p_^2^ |
| --- | --- | --- | --- | --- |
| Task Sequence | 1 | 0.04 | 0.85 | 0 |
| Sex | 1 | 0.19 | 0.66 | 0 |
| AlcoholExpect | 1 | 1.72 | 0.19 | 0.02 |
| AlcoholPharma | 1 | 0.15 | 0.70 | 0 |
| AlcoholExpect × Sex | 1 | 0.02 | 0.89 | 0 |
| AlcoholPharma × Sex | 1 | 1.67 | 0.20 | 0.02 |

*Note.* AlcoholExpect: Expectancy effect of alcohol, AlcoholPharma: Pharmacological effect of alcohol

**
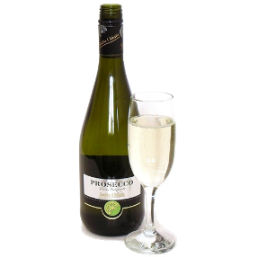
**
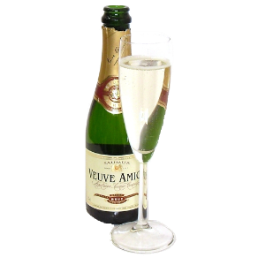

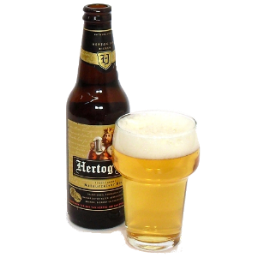

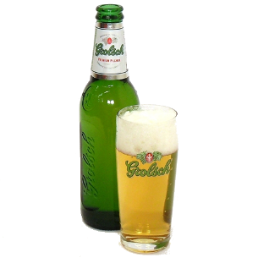

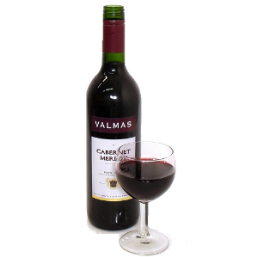


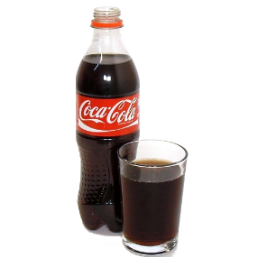

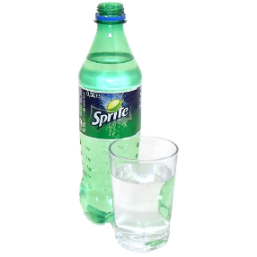

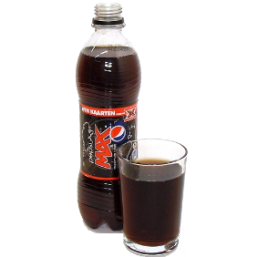

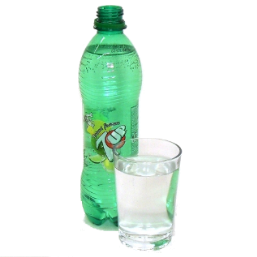

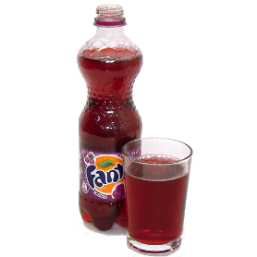


**Fig. S1.** Bottles used in the Chasing Bottles task

Source: Amsterdam Beverage Picture Set (Pronk, Deursen, Beraha, Larsen, & Wiers, 2015)


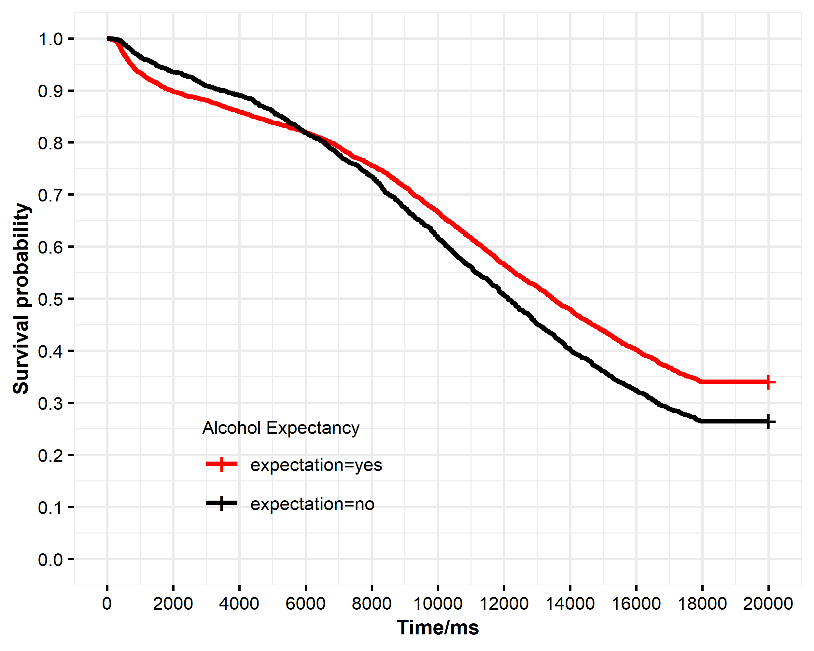


**Fig. S2a**. The expectancy effect of alcohol on the hazard rate over time


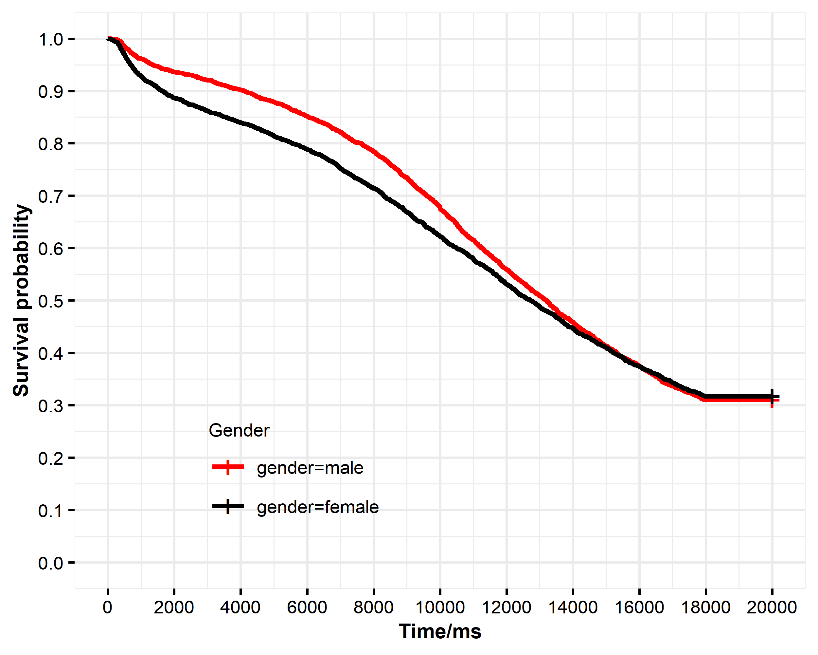


**Fig. S2b.** The effect of sex on the hazard rate over time


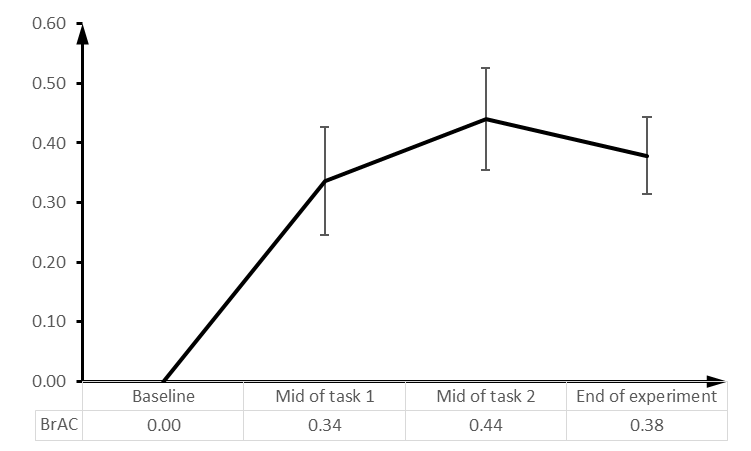


**Fig. S3.** Breath Alcohol Concentration readings at each time point


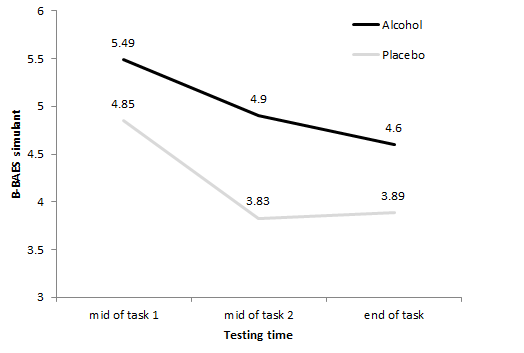


**Fig. S4a.** Simulant subscale of B-BAES for alcohol and placebo groups at different time points. For both groups, the simulant ratings declined significantly from the mid of task 1 to the mid of task 2. Though at each testing points the alcohol group felt more stimulant than the placebo group, this difference did not reach statistical significance


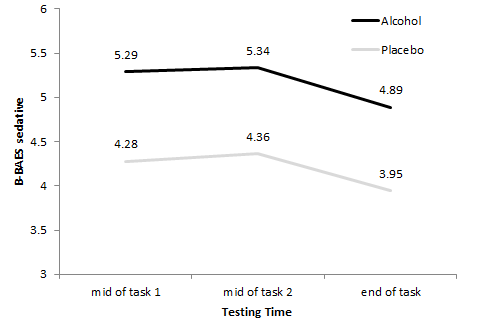


**Fig. S4b.** Sedative subscale of B-BAES for alcohol and placebo groups at different testing points. The alcohol group felt more sedative than the placebo group throughout the whole study. Also, there was a significant decline of sedative rating from the mid of task 2 to the end of task for both groups

**Short Clips:**

Neistat, C. Make it Count. (2012). Retrieved from

<https://www.youtube.com/watch?v=WxfZkMm3wcg> March 20, 2017.

It displayed a traveler’s ten-day trip around the world, during which he had a lot of adventures.

DreamWorksTV. Penguins of Madagascar. (2014). Retrieved from

<https://www.youtube.com/watch?v=AWxy9C5svFU> March 20, 2017.

It is the beginning of a cartoon. In a world of ice and snow, three penguins saved a penguin egg from their enemy’s mouth. The four penguins later acted as a family on their adventure tour.

**References**

Bartholow, B. D., Fleming, K. A., Wood, P. K., Cowan, N., Saults, J. S., Altamirano, L., … Sher, K. J. (2018). Alcohol effects on response inhibition: variability across tasks and individuals. *Experimental and Clinical Psychopharmacology*, *26*(3), 251–267. <https://doi.org/10.1037/pha0000190>

Campbell, A. E., Chambers, C. D., Allen, C. P. G., Hedge, C., & Sumner, P. (2017). Impairment of manual but not saccadic response inhibition following acute alcohol intoxication. *Drug and Alcohol Dependence*, *181*, 242–254. <https://doi.org/10.1016/j.drugalcdep.2017.08.022>

Caswell, A. J., Morgan, M. J., & Duka, T. (2013). Acute alcohol effects on subtypes of impulsivity and the role of alcohol-outcome expectancies. *Psychopharmacology*, *229*(1), 21–30. <https://doi.org/10.1007/s00213-013-3079-8>

Claes, L., Vertommen, H., & Braspenning, N. (2000). Psychometric properties of the Dickman Impulsivity Inventory. *Personality and Individual Differences*, *29*(1), 27–35. <https://doi.org/10.1016/s0191-8869(99)00172-5>

Conigrave, K. M., Hall, W. D., & Saunders, J. B. (1995). The AUDIT questionnaire: choosing a cut‐off score. *Addiction*, *90*(10), 1349–1356. <https://doi.org/10.1046/j.1360-0443.1995.901013496.x>

Courtney, K. E., Ashenhurst, J., Bacio, G., Moallem, N., Bujarski, S., Hartwell, E., & Ray, L. A. (2013). Craving and subjective responses to alcohol administration: validation of the desires for alcohol questionnaire in the human laboratory. *Journal of Studies on Alcohol and Drugs*, *74*(5), 797–802. http://doi.org/10.15288/jsad.2013.74.797

Dawe, S., & Loxton, N. J. (2004). The role of impulsivity in the development of substance use and eating disorders. *Neuroscience & Biobehavioral Reviews*, *28*(3), 343–351. <https://doi.org/10.1016/j.neubiorev.2004.03.007>

de Wit, H., Crean, J., & Richards, J. B. (2000). Effects of d-Amphetamine and ethanol on a measure of behavioral inhibition in humans. *Behavioral Neuroscience*, *114*(4), 830–837. http://doi.org/10.1037/0735-7044.114.4.830

Dougherty, D. M., Marsh-Richard, D. M., Hatzis, E. S., Nouvion, S. O., & Mathias, C. W. (2008). A test of alcohol dose effects on multiple behavioral measures of impulsivity. *Drug and Alcohol Dependence*, *96*(1–2), 111–120. <https://doi.org/10.1016/j.drugalcdep.2008.02.002>

Dougherty, D. M., Mullen, J., Hill-Kapturczak, N., Liang, Y., Karns, T. E., Lake, S. L., … Roache, J. D. (2015). Effects of tryptophan depletion and a simulated alcohol binge on impulsivity. *Experimental and Clinical Psychopharmacology*, *23*(2), 109–121. <https://doi.org/10.1037/a0038943>

Easdon, C. M., & Vogel-Sprott, M. (2000). Alcohol and behavioral control: impaired response inhibition and flexibility in social drinkers. *Experimental and Clinical Psychopharmacology*, *8*(3), 387–394. http://doi.org/10.1037/1064-1297.8.3.387

Fillmore, M. T., & Rush, C. R. (2002). Impaired inhibitory control of behavior in chronic cocaine users. *Drug & Alcohol Dependence*, *66*(3), 265–273. http://doi.org/10.1016/s0376-8716(01)00206-x

Fillmore, M. T., & Vogel-Sprott, M. (1999). An alcohol model of impaired inhibitory control and its treatment in humans. *Experimental and Clinical Psychopharmacology*, *7*(1), 49–55. http://doi.org/10.1037/1064-1297.7.1.49

Fillmore, M. T., & Vogel-Sprott, M. (2000). Response inhibition under alcohol: effects of cognitive and motivational conflict. *Journal of Studies on Alcohol*, *61(2)*, 239–246. http://doi.org/10.15288/jsa.2000.61.239

Gan, G., Guevara, A., Marxen, M., Neumann, M., Jünger, E., Kobiella, A., … Smolka, M. N. (2014). Alcohol-induced impairment of inhibitory control is linked to attenuated brain responses in right fronto-temporal cortex. *Biological Psychiatry*, *76*(9), 698–707. http://doi.org/10.1016/j.biopsych.2013.12.017

Guillot, C. R., Fanning, J. R., Bullock, J. S., McCloskey, M. S., & Berman, M. E. (2010). Effects of alcohol on tests of executive functioning in men and women: a dose response examination. *Experimental and Clinical Psychopharmacology*, *18*(5), 409–417. http://doi.org/10.1037/a0021053

Kareken, D. A., Dzemidzic, M., Wetherill, L., Eiler, W., Oberlin, B. G., Harezlak, J., … O’Connor, S. J. (2013). Family history of alcoholism interacts with alcohol to affect brain regions involved in behavioral inhibition. *Psychopharmacology*, *228*(2), 335–345. <https://doi.org/10.1007/s00213-013-3038-4>

Kramer, J. R., Chan, G., Hesselbrock, V. M., Kuperman, S., Bucholz, K. K., Edenberg, H. J., … Porjesz, B. (2010). A principal components analysis of the abbreviated Desires for Alcohol Questionnaire (DAQ). *Journal of Studies on Alcohol and Drugs*, *71*(1), 150–155. http://doi.org/10.15288/jsad.2010.71.150

Korucuoglu, O., Gladwin, T. E., & Wiers, R. W. (2015). Alcohol-induced changes in conflict monitoring and error detection as predictors of alcohol use in late adolescence. *Neuropsychopharmacology*, *40*(3), 614–621. http://doi.org/10.1038/npp.2014.209

Loeber, S., & Duka, T. (2009a). Acute alcohol decreases performance of an instrumental response to avoid aversive consequences in social drinkers. *Psychopharmacology*, *205*(4), 577–587. <https://doi.org/10.1007/s00213-009-1565-9>

Loeber, S., & Duka, T. (2009b). Acute alcohol impairs conditioning of a behavioural reward‐seeking response and inhibitory control processes—implications for addictive disorders. *Addiction*, *104*(12), 2013–2022. <https://doi.org/10.1111/j.1360-0443.2009.02718.x>

Loeber, S., & Duka, T. (2009c). Extinction learning of stimulus reward contingencies: the acute effects of alcohol. *Drug and Alcohol Dependence*, *102*(1–3), 56–62. <https://doi.org/10.1016/j.drugalcdep.2009.01.014>

Love, A., James, D., & Willner, P. (1998). A comparison of two alcohol craving questionnaires. *Addiction*, *93*(7), 1091–1102. http://doi.org/doi:10.1046/j.1360-0443.1998.937109113.x

McCarthy, D. M., Niculete, M. E., Treloar, H. R., Morris, D. H., & Bartholow, B. D. (2012). Acute alcohol effects on impulsivity: associations with drinking and driving behavior. *Addiction*, *107*(12), 2109–2114. http://doi.org/10.1111/j.1360-0443.2012.03974.x

Mulvihill, L. E., Skilling, T. A., & Vogel-Sprott, M. (1997). Alcohol and the ability to inhibit behavior in men and women. *Journal of Studies on Alcohol*, *58*(6), 600–605. http://doi.org/10.15288/jsa.1997.58.600

Nikolaou, K., Critchley, H., & Duka, T. (2013). Alcohol affects neuronal substrates of response inhibition but not of perceptual processing of stimuli signalling a stop response. *PLoS One*, *8*(9), e76649. <https://doi.org/10.1371/journal.pone.0076649>

Peacock, A., Cash, C., & Bruno, R. (2015). Cognitive impairment following consumption of alcohol with and without energy drinks. *Alcoholism: Clinical and Experimental Research*, *39*(4), 733–742. <https://doi.org/10.1111/acer.12680>

Plawecki, M. H., Windisch, K. A., Wetherill, L., Kosobud, A. E. K., Dzemidzic, M., Kareken, D. A., & O’Connor, S. J. (2018). Alcohol affects the P3 component of an adaptive stop signal task ERP. *Alcohol*, *70*, 1–10. <https://doi.org/10.1016/j.alcohol.2017.08.012>

Piasecki, T. M., Alley, K. J., Slutske, W. S., Wood, P. K., Sher, K. J., Shiffman, S., & Heath, A. C. (2012). Low sensitivity to alcohol: relations with hangover occurrence and susceptibility in an ecological momentary assessment investigation. *Journal of Studies on Alcohol and Drugs*, *73*(6), 925–932. <https://doi.org/10.15288/jsad.2012.73.925>

Presley, C. A. (1993). Alcohol and drugs on American college campuses. use, consequences, and perceptions of the campus environment. Volume I: 1989-91.

Pronk, T., Deursen, D. S., Beraha, E. M., Larsen, H., & Wiers, R. W. (2015). Validation of the Amsterdam Beverage Picture Set: a controlled picture set for cognitive bias measurement and modification paradigms. *Alcoholism: Clinical and Experimental Research*, *39*(10), 2047–2055. http://doi.org/doi:10.1111/acer.12853

Ray, L. A., Hart, E. J., & Chin, P. F. (2011). Self-Rating of the Effects of Alcohol (SRE): Predictive utility and reliability across interview and self-report administrations. *Addictive Behaviors*, *36*(3), 241–243. <https://doi.org/10.1016/j.addbeh.2010.10.009>

Reinert, D. F., & Allen, J. P. (2002). The Alcohol Use Disorders Identification Test (AUDIT): a review of recent research. *Alcoholism: Clinical And Experimental Research*, *26*(2), 272–279. http://doi.org/doi:10.1111/j.1530-0277.2002.tb02534.x

Reynolds, B., Richards, J. B., & de Wit, H. (2006). Acute-alcohol effects on the experiential discounting task (EDT) and a question-based measure of delay discounting. *Pharmacology Biochemistry and Behavior*, *83*(2), 194–202. https://doi.org/10.1016/j.pbb.2006.01.007

Spinola, S., Maisto, S. A., White, C. N., & Huddleson, T. (2017). Effects of acute alcohol intoxication on executive functions controlling self-regulated behavior. *Alcohol*, *61*, 1–8. <https://doi.org/10.1016/j.alcohol.2017.02.177>

Watson, D., & Clark, L. A. (1999). The PANAS-X: manual for the positive and negative affect schedule-expanded form.

Watson, D., Clark, L. A., & Tellegen, A. (1988). Development and validation of brief measures of positive and negative affect: The PANAS scales. *Journal of Personality and Social Psychology*, *54*(6), 1063–1070. http://doi.org/10.1037/0022-3514.54.6.1063

White, H. R., & Labouvie, E. W. (1989). Towards the assessment of adolescent problem drinking. *Journal of Studies on Alcohol*, *50*(1), 30–37. http://doi.org/10.15288/jsa.1989.50.30
